# Melanoma cell lysosome secretory burst neutralizes the CTL-mediated cytotoxicity at the lytic synapse

**DOI:** 10.1038/ncomms10823

**Published:** 2016-03-04

**Authors:** Roxana Khazen, Sabina Müller, Nicolas Gaudenzio, Eric Espinosa, Marie-Pierre Puissegur, Salvatore Valitutti

**Affiliations:** 1 Inserm, U1043, Toulouse F-31300, France; 2 CNRS, U5282, Toulouse 31300, France; 3 Université de Toulouse, UPS, Centre de Physiopathologie Toulouse-Purpan (CPTP), Toulouse F-31300, France; 4 Departement of Pathology, Institut Universitaire du Cancer-Oncopole de Toulouse, Toulouse 31059, France; 5 Present address: Department of Pathology, Stanford University School of Medicine, Stanford, California 94305, USA

## Abstract

Human melanoma cells express various tumour antigens that are recognized by CD8^+^ cytotoxic T lymphocytes (CTLs) and elicit tumour-specific responses *in vivo*. However, natural and therapeutically enhanced CTL responses in melanoma patients are of limited efficacy. The mechanisms underlying CTL effector phase failure when facing melanomas are still largely elusive. Here we show that, on conjugation with CTL, human melanoma cells undergo an active late endosome/lysosome trafficking, which is intensified at the lytic synapse and is paralleled by cathepsin-mediated perforin degradation and deficient granzyme B penetration. Abortion of SNAP-23-dependent lysosomal trafficking, pH perturbation or impairment of lysosomal proteolytic activity restores susceptibility to CTL attack. Inside the arsenal of melanoma cell strategies to escape immune surveillance, we identify a self-defence mechanism based on exacerbated lysosome secretion and perforin degradation at the lytic synapse. Interfering with this synaptic self-defence mechanism might be useful in potentiating CTL-mediated therapies in melanoma patients.

## Abstract (web summary)

Cytotoxic T lymphocytes recognise and eliminate tumour cells. Here, the authors show that on contact with these immune cells melanoma cells can resist T cell cytotoxicity by modulating the trafficking of their lysosomal compartment, this results in the degradation of the T cell protein perforin by the protease cathepsin B.

## Graphical abstract

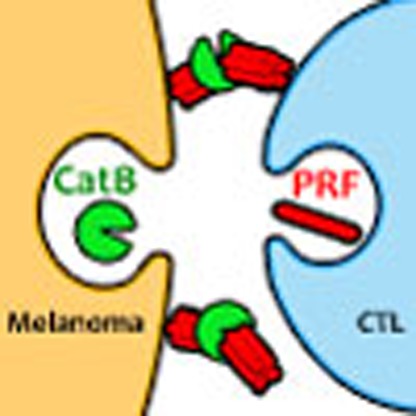

CD8^+^ cytotoxic T lymphocytes (CTLs) are major cellular effectors of the anti-tumour immune response^1^,^2^. They are, therefore, key components of therapeutic protocols aiming at potentiating immune response against cancer^3^. Clinical trials based on the induction of antigen-specific CTL responses against tumour cells have been reported in various types of cancer. Among those, melanoma is the most studied tumour type in terms of immune reactivity and experimental immunotherapy^4^,^5^. In melanoma patients, several strategies are currently being evaluated, including vaccination with dendritic cells carrying tumour antigens, adoptive transfer of tumour-specific CTL and treatment with immune-checkpoint inhibitors^5^,^6^,^7^,^8^,^9^. These strategies are mostly focused on strengthening CTL activation and effector function rather than on weakening tumour cell resistance to CTL attack. Although promising, those strategies are of limited efficacy and often present severe side effects^9^. Approaches aiming at dampening tumour cell resistance might therefore synergize with current therapeutic protocols and offer major benefits to patients.

A key pathway used by human CTL to kill their target cells is based on perforin/granzyme-mediated lethal hit delivery. Within minutes or seconds after productive T-cell receptor(TCR) engagement, the secretion of pore-forming protein perforin, granzyme B, and other proteases stored in CTL cytoplasmic granules (named lytic granules) takes place at the CTL/target cell lytic synapse^10^,^11^,^12^,^13^,^14^,^15^. Perforin-mediated penetration of granzyme B into target cells triggers an apoptotic cascade leading to target cell death^16^,^17^. Although CTLs are equipped with other mechanisms of cytotoxicity, the perforin/granzyme pathway is key for human CTL and natural killer cell effector function, as indicated by the immunodeficiency status and the alteration of immune homeostasis in patients with genetic mutations of perforin or of molecules implicated in lytic granule docking and fusion to plasma membrane^18^,^19^. In spite of the major interest in potentiating CTL responses in melanoma patients, the molecular dynamics of perforin-mediated lethal hit delivery in this context remains to be elucidated. In particular, while it is well known that melanoma cells possess resistance mechanisms downstream of early pro-apoptotic granzyme B activity^20^,^21^, whether melanoma cells are endowed with mechanisms interfering with the early steps of perforin-mediated pore formation at the lytic synapse is presently elusive.

In this work, we attempted to answer this outstanding question by monitoring the melanoma cell side of the lytic synapse during the encounter with cognate CTL, under conditions in which lethal hit delivery by CTL is efficiently triggered. Our results show that melanoma cells rapidly respond to CTL at the lytic synapse by a secretory burst of lysosome/late endosomes (LLE). Importantly, this leads to cathepsin-mediated degradation of perforin. Inhibition of this melanoma response by different means impairs melanoma cell resistance to perforin-mediated cytotoxicity. Our results reveal a mechanism of perforin pathway inactivation, which might contribute to melanoma cell immune resistance. They may inspire new therapeutic approaches complementary to the immuno-modulatory strategies being currently used for the treatment of melanoma patients.

## Results

### Lethal hit deficiency at the CTL/melanoma cell lytic synapse

We have previously shown that human CTL interacting *in vitro* with antigen-pulsed melanoma cells are efficiently triggered to lytic granule secretion, yet melanoma cells can resist for prolonged time to CTL-mediated cytoxicity^22^. To dissect the molecular mechanisms of melanoma cell resistance to CTL attack at the lytic synapse, we used, as cellular model, melanoma cells that were either pulsed with strong antigenic ligands (peptides of the human cytomegalovirus protein pp65) or left unpulsed before conjugation with cognate CTL. This strategy was chosen to focus on the melanoma cell side of the lytic synapse in conditions in which optimal CTL activation was ensured. Virus specific CTL are indeed fully activated to lethal hit delivery when interacting with peptide-pulsed melanoma cells^22^.

As target cells we employed the metastatic melanoma cell line D10 that we have previously characterized for its sustained resistance to CTL-mediated cytotoxicity^22^ and JY cells, an Epstein–Barr virus (EBV)-transformed B-cell line largely employed as a conventional target cell for human CTL^23^. D10 cells have been recently characterized for their high clonogenic capacity and for their capacity to grow in spheroids^24^.

**Figure 1:**
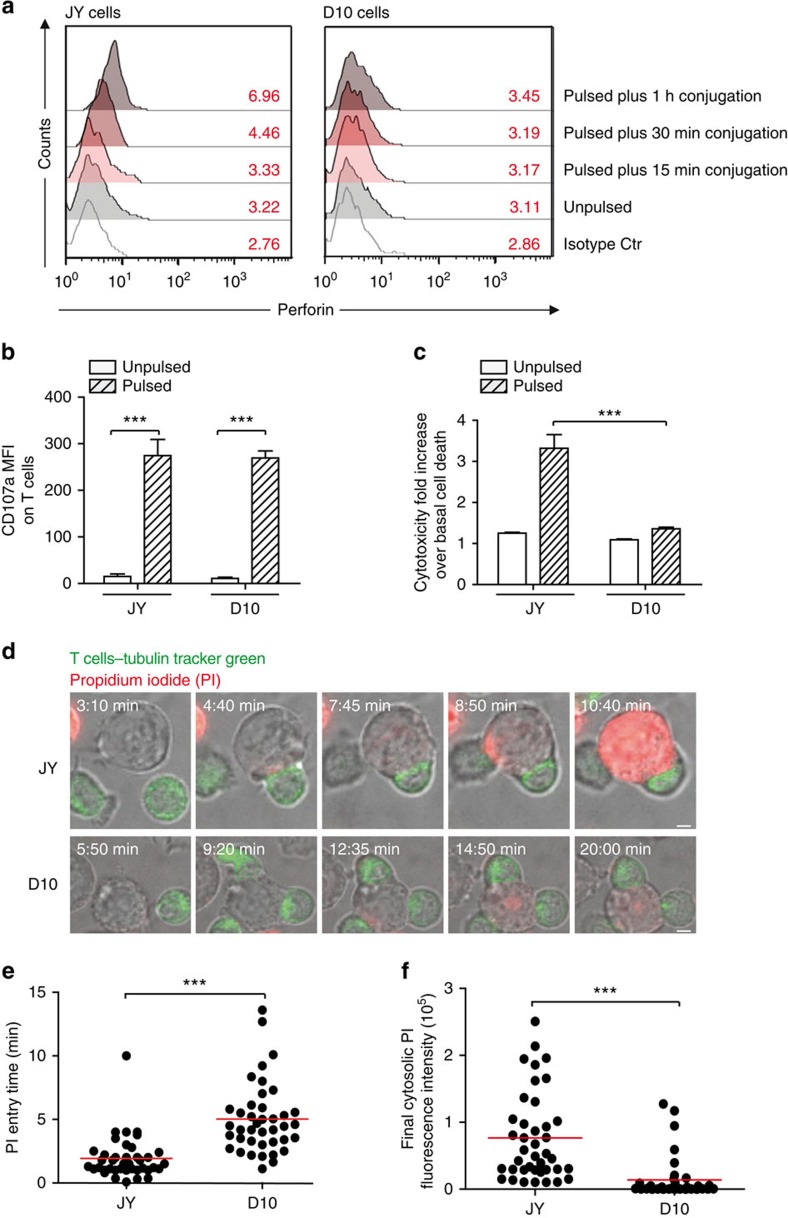
Defective lethal hit delivery at the CTL/melanoma cell synapse. (**a**) Time kinetics of perforin staining on the surface of conventional target cells (JY) and melanoma cells (D10) following conjugation with CTL. Target cells were either unpulsed or pulsed with 10 μM antigenic peptide. The median fluorescence intensity (MFI) of perforin staining is indicated. (**b**) Efficient activation of CTL to lethal hit delivery following encounter with peptide-pulsed melanoma cells. Target cells either pulsed or not with antigenic peptide were conjugated for 1 h with CTL. Cells were stained for CD107a extracellular exposure at 4 °C. (**c**) Measurement of target cell killing. Cytotoxicity was evaluated by FACS analysis in target cells either pulsed or not with antigenic peptide following 4 h incubation with CTL. Cytotoxicity is expressed as fold increase over corresponding basal death. (**d**) Sequences of snapshots depicting either JY or D10 cells pulsed with the antigenic peptide interacting with tubulin tracker green-labelled CTL. PI was added to the culture medium to a final concentration of 200 μM at time 0. Perforin pore formation on target cells was detected by PI internalization (red). Typical conjugates with the JY and D10 target cells are shown. Supplementary Movies 1 and 2 show the entire time-lapse recording. Scale bars, 5 μm. (**e**) Time required for detection of initial PI entry in target cells in JY or D10/CTL conjugates as detected by 1 h time-lapse confocal microscopy. Red bars represent the mean time. (**f**) Measurement of the cytosolic PI intensity in target cells at the end of the time-lapse video recording in CTL/target cell conjugates. Red bars represent the mean PI fluorescence intensity. Analysis in **e** and **f** was performed only on cells exhibiting detectable PI entry. In **a**, results are representative of three independent experiments. In **b** and **c** results are expressed as mean±s.e.m. of three independent experiments (**b**) and four independent experiments (**c**). In **e** and **f** data are from 40 conjugates for each cell type. Data are from three independent experiments. Unpaired Student's *t*-test using the GraphPad Prism software (version 6; GraphPad) was used to determine the statistical significance of differences between the groups. ^***^P<0.001, ^**^P<0.01.

To investigate whether melanoma cells might impair early steps of CTL-mediated cytotoxicity, we assessed, in a first approach, perforin staining on target cell surface following short-time interaction with CTL. As shown in Fig. 1a and Supplementary Fig. 1, melanoma cells exhibited a limited perforin staining when compared to conventional target cells, although CTL were similarly activated to lethal hit delivery during interaction with the two different target cell types, as revealed by the increase of surface CD107a expression (Fig. 1b and Supplementary Fig. 2). Under these experimental conditions melanoma cells exhibited resistance to CTL-mediated cytotoxicity when compared with conventional target cells, in line with our previously reported data (Fig.1c and ref. 22). Deficient perforin staining was also observed in five additional metastatic melanoma cell lines (Supplementary Fig. 3).

To better characterize this phenomenon, we investigated whether the observed defective staining of perforin on melanoma cell surface would translate into an impaired pore formation. To this end, the efficacy and time kinetics of lethal hit delivery to individual melanoma cells were studied by time-lapse confocal laser scanning microscopy. Propidium Iodide (PI) was added at high concentration to the culture medium to monitor lethal hit transmission based on the entry of this probe via the pores formed on perforin binding on target cell surface^25^.

CTL/melanoma cell conjugates from four independent experiments were analysed to define the intensity of PI staining and the time elapsed between the initial CTL/target cell contact and the appearance of the PI staining at the target cell synaptic area. Results were compared with those obtained with conventional target cells. This analysis showed that the initial entry of PI was delayed in melanoma cells when compared with conventional target cells (Fig. 1d,e and Supplementary Movies 1 and 2). Moreover, melanoma cells exhibited an overall lower PI staining (Fig. 1d,f and Supplementary Movies 1 and 2).

**Figure 2:**
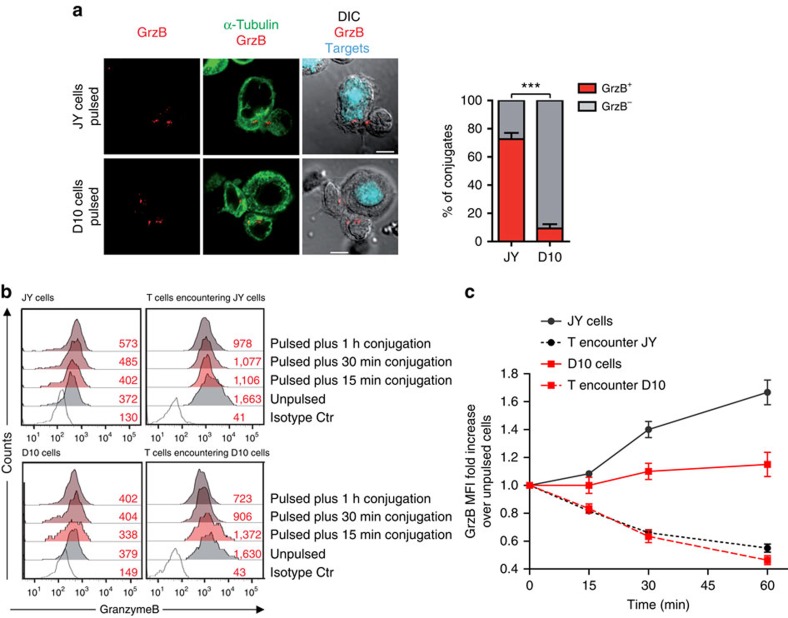
Defective granzyme B penetration in melanoma cells. (**a**) Visualization of GrzB staining in CTL/target cell conjugates by confocal laser scanning microscopy. Target cells were previously pulsed with 10 μM peptide. Cells were stained with anti-α tubulin (green) and anti GrzB (red). Target cells were identified by loading them with Cell Tracker Blue before conjugation (cyan). Percentage of GrzB^+^ target cells in peptide-pulsed JY and D10 cells are indicated. Sixty conjugates from three independent experiments were scored. Scale bars, 5 μm. Results are expressed as mean±s.e.m. of three independent experiments. Unpaired Student's *t*-test using the GraphPad Prism software was used to determine the statistical significance of differences between the groups. ^***^P<0.001. (**b**) Left panels: time kinetics of GrzB staining in conventional target cells (JY) and melanoma cells (D10), either unpulsed or pulsed with 10 μM peptide concentration, following conjugation with CTL. Right panels: time course of GrzB loss in CTL following interaction with target cells. Target cells were either unpulsed or pulsed with the antigenic peptide. The median fluorescence intensity of GrzB staining is indicated. Results are from one representative experiment out of three. (**c**) Pooled results from three independent experiments showing the time-dependent loss of GrzB by CTL interacting with JY cells (black dotted lines) or with D10 cells (red dotted lines) and GrzB uptake by JY cells (black plain lines) or by D10 cells (red plain lines). MFI, median fluorescence intensity. Results are expressed as mean±s.e.m. of three independent experiments.

In a third approach aiming at defining whether a defective transmission of lytic enzymes might occur at the CTL/melanoma cell synapse, we visualized granzyme B (GrzB) staining in target cells 15 min after conjugation with CTL using confocal laser scanning microscopy. This analysis showed that following interaction with CTL, while GrzB staining was significantly detected in a large fraction of sensitive target cells (∼73%), only a small fraction of melanoma cells were detected GrzB^+^ (∼8%) (Fig. 2a). Defective GrzB penetration in melanoma cells, when compared with sensitive target cells, was also measured by fluorescence-activated cell sorting (FACS) analysis in fixed and permeabilized CTL/target cell conjugates (Fig. 2b,c). This analysis allowed us to show that GrzB release by CTL is similarly triggered following interaction with melanoma cells as compared with conventional target cells, thus ruling out the possibility that defective GrzB transfer would result from defective CTL activation (Fig. 2b,c).

Taken together, the above results point out a deficient lethal hit delivery at the CTL/melanoma cell lytic synapse characterized by altered perforin pore formation and GrzB internalization.

### High-rate LLE vesicle trafficking in melanoma cells

**Figure 3:**
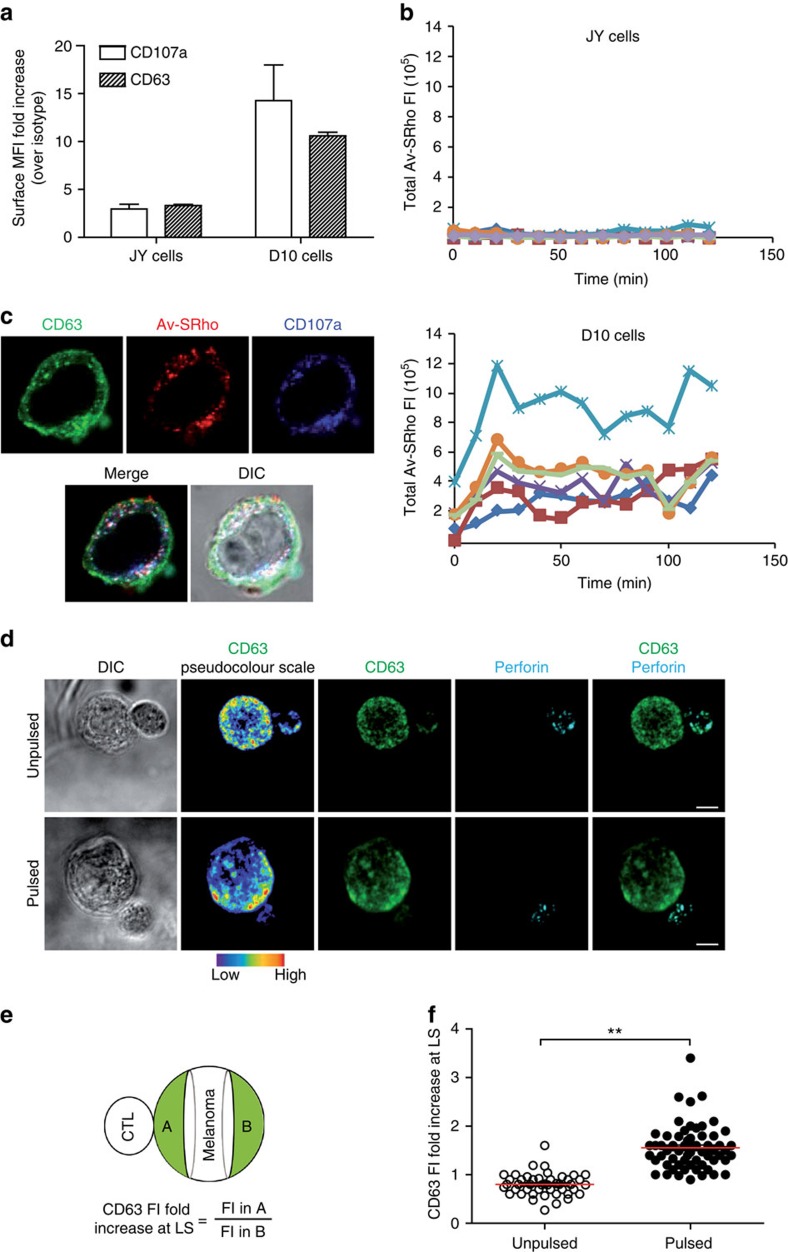
Melanoma cells exhibit a high-rate vesicular trafficking and enrich LLE vesicles at the lytic synapse during conjugation with CTL. (**a**) Extracellular staining for CD107a and CD63 on JY and D10 cell surface as detected by FACS analysis. Results are shown as median fluorescence intensity (MFI) fold increase over isotype control. Results are expressed as mean±s.e.m. of five independent experiments. (**b**) basal lysosomal endo/exocytosis was quantified via Av-SRho uptake by JY (upper panel) or D10 cells (lower panel) in the presence of 8 μg ml^−1^ Av-SRho in the culture medium. Panels show Av-SRho fluorescence intensity in cells during 2 h acquisition, as measured by time-lapse confocal microscopy. Graphs show six cells representative of the two cell types from three independent experiments. (**c**) Av-SRho accumulates in melanoma cells lysosomal compartment. Melanoma cells were incubated in the presence of 8 μg ml^−1^ of Av-SRho (red) overnight. Cells were fixed, permeabilized and stained for CD107a (blue) and CD63 (green). (**d**) Melanoma cells either unpulsed or pulsed with 10 μM antigenic peptide were conjugated with peptide-specific CTL for 5 min at 37 °C. Cells were then fixed, permeabilized and stained for CD63 (green and pseudo-colour) and perforin (cyan). Scale bars, 5 μm. (**e**) Scheme depicting the gating strategy used to measure CD63 fluorescence intensity in melanoma cells in a cell volume corresponding to the synaptic area and in an equal volume drawn at the opposite side of the cell. (**f**) Re-localization of lysosomal compartments was quantified by measuring the CD63 fluorescence intensity fold increase at the lytic synapse in melanoma cells (as indicated in the scheme). In all, 45 and 60 conjugates (for unpulsed and peptide-pulsed cells, respectively) formed by only one melanoma cell and one CTL were scored. Data are from three independent experiments. Bars indicate mean values. Unpaired Student's *t*-test using the GraphPad Prism software was used to determine the statistical significance of differences between the groups. ^**^P<0.01.

It is well established that LLE play a key role in cell membrane repair following physical, chemical and biological assaults^26^,^27^. We thus investigated the dynamics of melanoma late LLE as compared with those of conventional target cells susceptible to CTL-mediated cytotoxicity. FACS analysis showed that melanoma cells exhibited higher constitutive CD107a and CD63 surface expression when compared with conventional target cells, suggesting that melanoma cells might exhibit a constitutively active secretion of LLE vesicles (Fig. 3a). We therefore attempted to monitor the constitutive exocytosis and recycling of LLE on melanoma cell surface using time-lapse microscopy.

To do so, we took advantage of a method we recently set-up to visualize real-time granule exocytosis by human mast cells based on the addition of avidin-sulforhodamine (Av-SRho) to culture medium, which binds to the serglycin proteoglycans exposed on cell surface on granule exocytosis^28^. Although serglycin-proteoglycan is best known as a hematopoietic cell granule proteoglycan, it is also expressed in human endothelial cells and in some metastatic tumours^29^,^30^. Accordingly, our data show that it is expressed by both conventional target cells and melanoma cells (Supplementary Fig. 4). We thus monitored, by time-lapse microscopy, the constitutive exocytosis of LLE vesicles in melanoma cells by adding Av-SRho to the culture medium. This analysis revealed a constitutive exposure of lysosomal vesicles on melanoma cell surface. In comparison, the conventional target cells failed to display detectable constitutive exposure of lysosomal vesicles on their surface (Fig. 3b; Supplementary Movies 3 and 4).

In parallel experiments, overnight incubation of melanoma cells with Av-SRho resulted in its uptake. Intracellular Av-SRho partially co-localized with CD107a and CD63 within intracellular vesicles, validating the use of Av-SRho as a tool to study melanoma LLE exposure (Fig. 3c).

Taken together, the above results reveal that melanoma cells exhibit a constitutive high-rate LLE vesicle exocytosis at the cell surface.

### Melanoma cell LLE vesicles exocytosis towards attacking CTL

We next investigated whether vesicular trafficking in melanoma cells might be exacerbated at lytic synapses formed with cognate CTL. In a first approach, we investigated, by confocal microscopy the localization of CD63^+^ intracellular vesicles in fixed and permeabilized CTL/melanoma cell conjugates. As shown in Fig. 3d, peptide-pulsed melanoma cells engaged in cognate interactions with CTL, displayed a significant enrichment of CD63 towards the lytic synapse. Such enrichment was not observed in non-cognate interactions (unpulsed melanoma cells). Morphological data were quantified by drawing two equal regions, one on the melanoma cell side of the lytic synapse and the other on the opposite side of the cell (see scheme in Fig. 3e). Three-dimensional (3D) measurements of the CD63 fluorescence intensity (FI) in the cell volume defined by the regions were performed (see Methods). This analysis provided quantitative evidence of an antigen-dependent enrichment of melanoma cell CD63^+^ endosomal compartment at the synaptic area in a significant number of CTL/melanoma cell conjugates (Fig. 3f).

**Figure 4:**
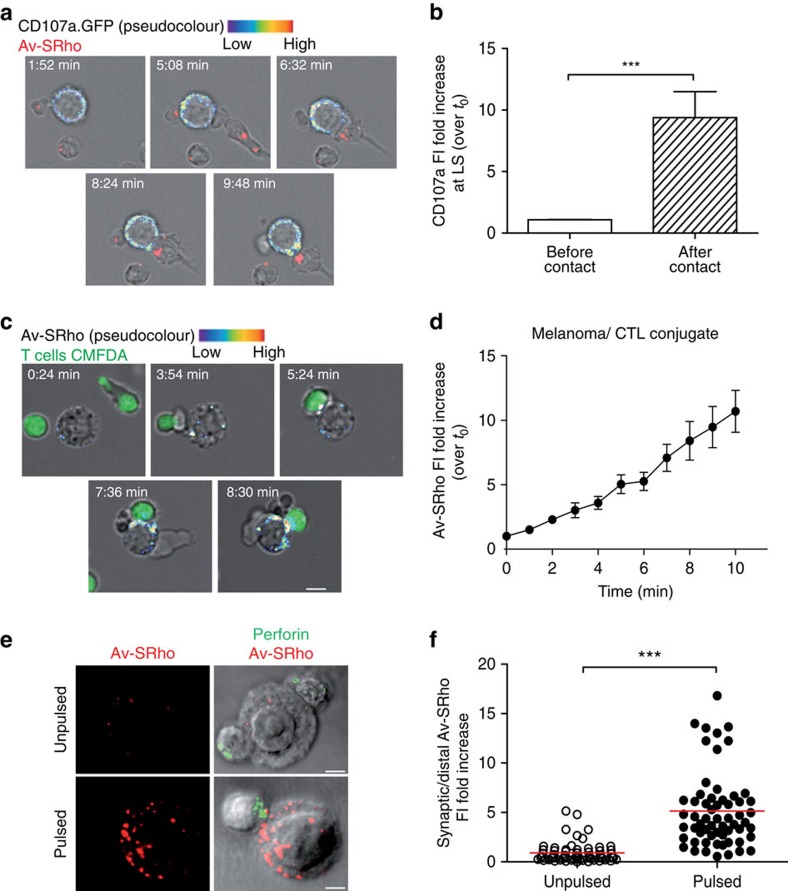
Recruitment of melanoma cell lysosomal compartment to the lytic synapse. (**a**,**b**) Melanoma cells expressing CD107a-GFP and pulsed with 10 μM antigenic peptide were monitored during interaction with CTL using a spinning-disk confocal microscope. (**a**) Snap shots depicting the CD107a-GFP^+^ LLE localization before conjugation and after conjugation. The pseudo colour scale indicates molecular enrichment. Snapshots are from Supplementary Movie 5. (**b**) Quantification of CD107a fluorescence intensity (FI) fold increase. FI either before or 5 min after cell–cell conjugation in 13 melanoma cells conjugated with CTL was measured at the lytic synapse and normalized over FI measured in the region of interest at time 0. Data are from five independent experiments. (**c**,**d**) Peptide-pulsed melanoma cells were monitored during interaction with CTL in the presence of 8 μg ml^−1^ Av-SRho by spinning-disk confocal microscopy. (**c**) Sequences of snap shots depicting enhanced LLE vesicle exposure in peptide-pulsed melanoma cells during conjugation with CMFDA loaded CTL (green). Lysosomal exposure is detected via Av-SRho uptake (pseudo colour scale intensity). Snapshots are from Supplementary Movie 6. (**d**) Measurement of Av-SRho FI fold increase in peptide-pulsed melanoma cells during conjugation with CTL. Intensities were scored from 14 conjugates in nine independent experiments and normalized over Av-SRho FI of the cell of interest at time 0. Data are expressed as mean±s.e.m. of the scored cells. (**e**) Melanoma cells either pulsed or not with antigenic peptide were conjugated with peptide-specific CTL for 5 min at 37 °C in the presence of Av-SRho. Cells were fixed, permeabilized and stained for perforin (green). (**f**) Exposure of LLE vesicles was quantified by measuring the Av-SRho FI fold increase at the lytic synapse in melanoma cells (as indicated in the scheme in Fig. 3e). In all, 61 and 60 conjugates (for unpulsed and peptide-pulsed cells, respectively) formed by one melanoma cell and one CTL were scored. Data are from three independent experiments. Bars indicate mean values. Scale bars, 5 μm. Unpaired Student's *t*-test using the GraphPad Prism software was used to determine the statistical significance of differences between the groups. ^***^P<0.001.

To monitor the dynamics of the LLE in melanoma cells facing the attack of cognate CTL, we expressed CD107a/GFP in melanoma cells. CD107a/GFP^+^ intracellular vesicles were visualized by time-lapse microscopy using a spinning-disk confocal microscope. On contact with CTL, peptide-pulsed melanoma cells displayed a rapid re-localization of the CD107a/GFP^+^ compartment as measured by an accumulation of GFP FI at the synaptic area during the first minutes after conjugation with CTL (Fig. 4a,b and Supplementary Movie 5). To monitor CTL lytic granule polarization towards target cells with the dynamics of melanoma LLE on cognate cell–cell interaction in T cell/melanoma cell conjugates, we took advantage of the observation that pretreatment of CTL with either Av-SRho or Av-Alexa488 allows to stain lytic granules (as shown by Av-SRho and perforin co-localization in fixed and permeabilized CTL, Supplementary Fig. 5). We could therefore monitor CTL lytic granule dynamics (as detected by the polarization of Av-SRho loaded lytic granules) together with melanoma cell LLE polarization by time-lapse microscopy as indicated in Fig. 4a.

Having observed that melanoma cells rapidly re-locate their LLE towards the contact site formed with cognate CTL, we asked whether the high rate of constitutive lysosomal secretion observed in melanoma cells might be further enhanced on contact with cognate CTL. We thus visualized the exposure of lysosomal vesicles on peptide-pulsed melanoma cell surface during interaction with CTL by adding Av-SRho to the culture medium. As shown in Fig. 4c,d and Supplementary Movie 6, melanoma cells exhibited an increased staining with Av-SRho during the first 10 min after contact with CTL. In contrast, unpulsed melanoma cells did not undergo detectable increase of Av-SRho binding on contact with CTL (Supplementary Movie 7). We also monitored CTL lytic granule polarization towards target cells in parallel with the secretory burst of melanoma LLE on cognate cell–cell interaction. Dynamics of CTL lytic granules was visualized by monitoring the polarization of Av-Alexa488-loaded lytic granules; whereas, melanoma cell LLE exposure was detected by the addition of Av-SRho to the culture medium. This analysis extended the above results by showing that the melanoma cell LLE exocytosis occurs following CTL lytic granule re-polarization (Supplementary Movie 8).

To quantify this phenomenon in a large number of melanoma cells, we analysed fixed CTL/melanoma cell conjugates after 5 min co-culture in the presence of Av-SRho. As shown in Fig. 4e in unpulsed conditions perforin^+^ lytic granules of CTL did not polarize towards melanoma cells and melanoma cell exhibited non-polarized basal level of Av-SRho binding. Conversely, when melanoma cells were previously pulsed with the antigenic peptide, CTL polarized their lytic granules towards melanoma cells and melanoma cells exhibited a strong Av-SRho binding that was enriched at the synaptic area. Morphological data were quantified by drawing two equal regions, one on the melanoma cell side of the lytic synapse and the other on the opposite side of the cell (as indicated in Fig. 3e). 3D measurements of the Av-SRho FI in the cell volume defined by the regions showed an increase of Av-SRho staining in the synaptic volume in the majority of peptide-pulsed melanoma cells (Fig. 4f). It should be noted that conventional target cells exhibited a barely detectable exposure of LLE vesicle when interacting with cognate CTL in the presence of Av-SRho (Supplementary Movies 9 and 10).

Taken together, the above results show that melanoma cells respond to CTL attack by adjusting the direction of their vesicular trafficking towards the lytic synapse and by enhancing global late-endosomal vesicle exocytosis on the cell surface.

### Melanoma cells degrade perforin from CTL granules

Having observed that melanoma cells activate a late-endosomal vesicle exocytosis process towards cognate CTL, we asked whether this mechanism might neutralize CTL-mediated cytotoxicity by degrading critical lytic molecules. We investigated the possibility that perforin might be degraded by melanoma cells since this enzyme has been previously reported to be substrate of lysosomal hydrolase cathepsin B (CatB)^31^,^32^, and since human and mouse melanoma cells are known to express high levels of cathepsin B^33^. In line with this hypothesis, the level of exposure of the CatB on melanoma cell surface on CTL attack was significantly higher than that on conventional target cell surface (Supplementary Fig. 6).

**Figure 5:**
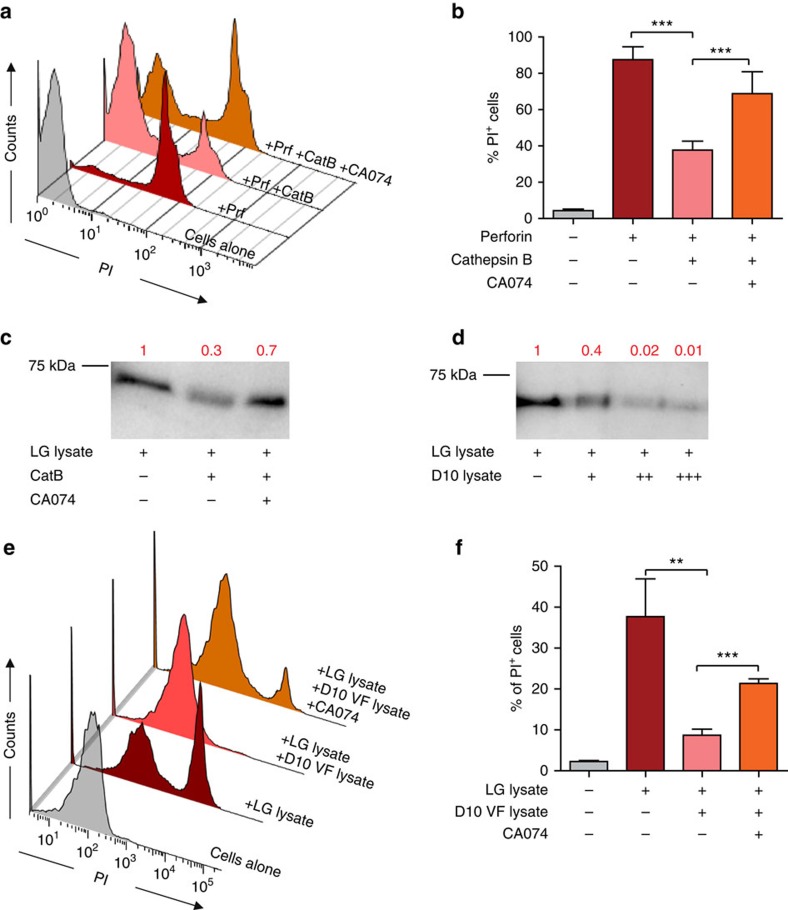
Perforin degradation by cathepsin and melanoma cell extracts. (**a**,**b**) Measurement of perforin lytic activity on Jurkat cells. Jurkat cells were incubated with purified human perforin either in the absence or in the presence of CatB or of CatB plus CA074 for 1 h. PI entry in cells was measured by flow cytometry. (**a**) Plots show results from one representative experiment; (**b**) data are expressed as mean±s.e.m. of four independent experiments. (**c**,**d**) Degradation of perforin in isolated CTL lytic granules. (**c**) Western blot analysis of lytic granule perforin either untreated or incubated with CatB or with CatB plus CA074. (**d**) Western blot analysis of lytic granule perforin either untreated or incubated with increasing concentrations of melanoma cell lysates (+ 0.5 μg ml^−1^; ++ 1 μg ml^−1^; +++ 3 μg ml^−1^). Results are from one representative experiment out of three. Numbers indicate band intensity fold increase. (**e**,**f**) Jurkat cells were incubated for 1 h with purified human lytic granules lysate either in the absence or in the presence of melanoma cell vesicular fraction lysates or of melanoma cell vesicular fraction lysates plus CA074 for 1 h. PI entry in cells was measured by flow cytometry. (**e**) Plots show result from one representative experiment; (**f**) data are expressed as mean±s.e.m. of three independent experiments. Unpaired Student's *t*-test using the GraphPad Prism software was used to determine the statistical significance of differences between the groups. ^***^P<0.001, ^**^P<0.01. LG, lytic granules; VF, vesicular fraction.

In a first approach, we employed FACS analysis to test whether CatB might inhibit perforin lytic activity. Perforin-sensitive Jurkat cells were incubated with purified human perforin either in the absence or in the presence of CatB or in the presence of CatB together with its inhibitor CA074. Incubation of Jurkat cells with purified perforin resulted in a strong cell permeabilization (as detected by PI entry) that was inhibited by CatB. Perforin lytic function was recovered in the presence of CA074 (Fig 5a,b).

In a second approach, we investigated cathepsin-mediated degradation of perforin contained in CTL lytic granules by western blot analysis. Incubation of lytic granule extracts with CatB resulted in perforin degradation. This degradation was inhibited by the addition of CA074 (Fig. 5c; Supplementary Fig. 7). Interestingly, dose-dependent lytic granule perforin degradation was also observed when lytic granule extracts were co-incubated with melanoma cell lysates (Fig. 5d; Supplementary Fig. 7).

In a further approach, we incubated CTL lytic granule lysates with Jurkat cells in the presence of a purified vesicular fraction of melanoma cells. This analysis showed that melanoma cell vesicular fraction was able to inhibit the lytic activity of CTL granules. Lytic activity was recovered in the presence of CA074 (Fig. 5e,f).

Taken together the above results show that melanoma cells are endowed of an intrinsic capacity to degrade perforin.

### SNAP-23 silencing in melanoma cells enhances cytotoxicity

**Figure 6:**
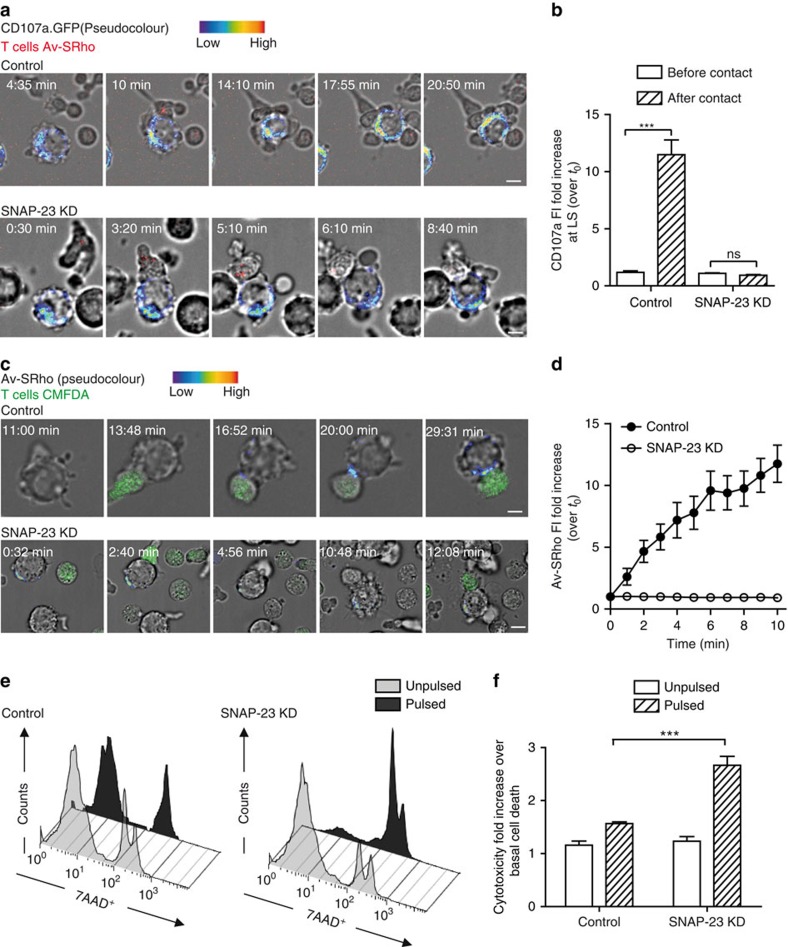
Interference with melanoma cell vesicular trafficking enhances their susceptibility to perforin-mediated cytotoxicity. (**a**) Upper panel: sequence of snap shots depicting dynamics of CD107a/GFP^+^ intracellular vesicles (pseudo colour scale intensity) in control non-targeting shRNA (shCtr) transfected 10 μM peptide-pulsed melanoma cell during conjugation with CTL. Snapshots are from Supplementary Movie 13. Lower panel: sequence of snap shots depicting dynamics of CD107a/GFP^+^ intracellular vesicles (pseudo colour scale intensity) in SNAP-23 silenced peptide-pulsed melanoma cell during its conjugation with a CTL. Snapshots are from Supplementary Movie 11. CTL lytic granules are stained in red. (**b**) Quantification of CD107a fluorescence intensity (FI) fold increase at the lytic synapse of 8 SNAP-23 silenced peptide-pulsed melanoma cells conjugated with CTL either before or 5 min after cell–cell conjugation. Data are from two independent experiments. (**c**) Upper panel: sequence of snap shots depicting LLE vesicle exocytosis of shCtr transfected peptide-pulsed melanoma cells interacting with CMFDA-loaded CTL (green). Lysosomal exposure is detected via Av-SRho binding (pseudo colour scale intensity). Snapshots are from Supplementary Movie 14. Lower panel: sequence of snap shots depicting LLE vesicle exocytosis of a SNAP-23 silenced peptide-pulsed melanoma cell interacting with CMFDA loaded CTL (green). Lysosomal exposure is detected via Av-SRho binding (pseudo colour scale intensity). Snapshots are from Supplementary Movie 12. (**d**) Measurement of Av-SRho FI fold increase in SNAP-23 silenced peptide-pulsed melanoma cells during conjugation with CTL. Intensities were scored from eight conjugates in three independent experiments. Data are expressed as mean±s.e.m. of the scored cells. (**e**,**f**) Cytotoxicity was evaluated in melanoma cells either transduced with shCtr or with shRNA targeting SNAP-23 (shSNAP-23) following 4 h incubation with CTL. Melanoma cells were either unpulsed or peptide-pulsed and cytotoxicity was quantified by measuring 7-AAD uptake by FACS analysis. (**e**) A representative experiment is shown; (**f**) pooled data from three independent experiments. Cytotoxicity is expressed as fold increase over corresponding basal death in transduced cells. Scale bars, 5 μm. Unpaired Student's *t*-test using the GraphPad Prism software was used to determine the statistical significance of differences between the groups. ^***^P<0.001.

To establish whether a mechanistic link might exist between the polarized LLE exocytosis of melanoma cells and the defective perforin-mediated cytotoxicity, we devised strategies to interfere with melanoma cell lysosomal compartment dynamics and function. The expression of SNAP-23, a SNARE molecule known to mediate lysosomal vesicle exocytosis^34^,^35^ was silenced in melanoma cells by transfection of a specific shRNA. This treatment resulted in the reduction of SNAP-23 expression in melanoma cells of 60–70%, as detected by polymerase chain reaction and by antibody staining (Supplementary Fig. 8). We tested whether the reduced SNAP-23 expression would interfere with the dynamics and secretion of LLE compartments in melanoma cells. The dynamics of CD107a/GFP^+^ intracellular vesicles in cells treated with SNAP-23 shRNA was profoundly altered. Indeed, the rapid re-localization of the CD107a/GFP^+^ compartment towards the CTL was abrogated and no enrichment of the GFP FI at the synaptic area could be measured (Fig. 6a,b; Supplementary Movie 11).

Accordingly, when we monitored the exposure of LLE vesicles (as detected by the addition of Av-SRho to the culture medium) on the surface of melanoma cells with reduced SNAP-23 expression, a profound inhibition of endosomal vesicle exocytosis was observed, as detected by a lower Av-SRho uptake (Fig. 6c,d; Supplementary Movie 12). Transfection of melanoma cells with control shRNA did not affect CD107a/GFP^+^ vesicle synaptic enrichment nor LLE exocytosis (Fig. 6a–d; Supplementary Movies 13, 14 and 15).

We next investigated the impact of SNAP-23 silencing on melanoma cell resistance to CTL attack. As shown in Fig. 6e,f, reducing the expression of SNAP-23 resulted in increased susceptibility to CTL-mediated cytotoxicity.

Taken together, these results establish a mechanistic link between active lysosomal secretion in melanoma cells and defective perforin-mediated cytotoxicity.

### Enhanced cytotoxicity by perturbing lysosome function

**Figure 7:**
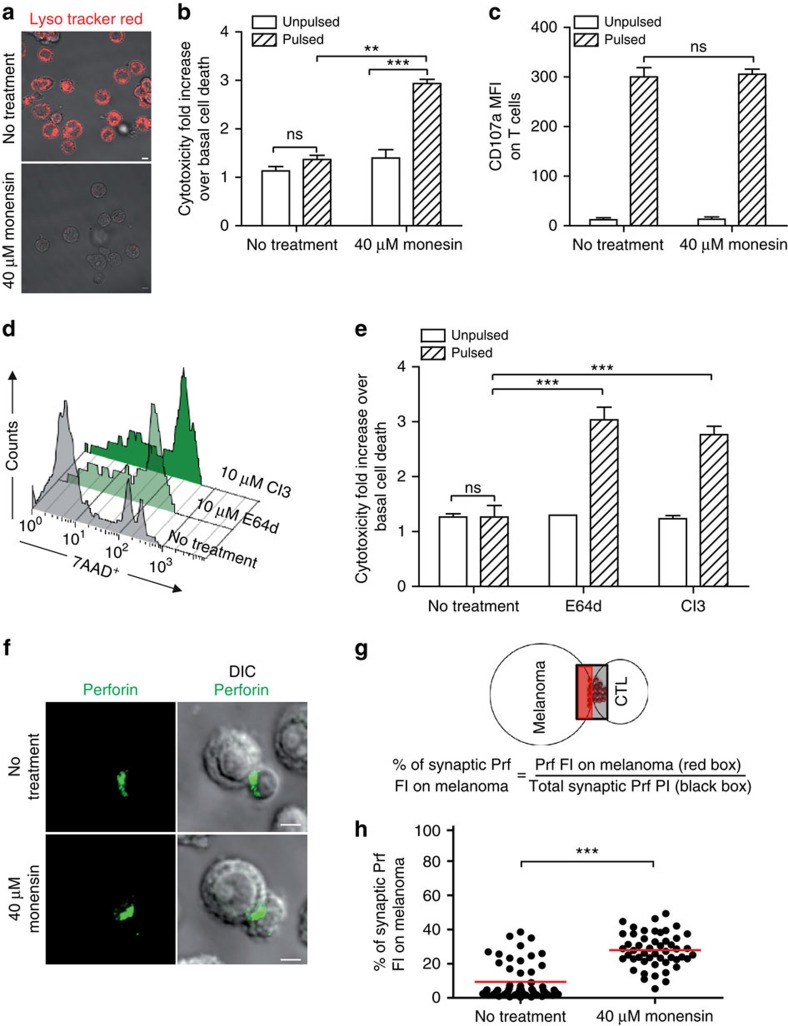
Impairment of melanoma cell lysosome function enhances their susceptibility to perforin-mediated cytotoxicity. (**a**) Cells were either untreated or treated for 2 h with 40 μM monensin, washed and stained with lysotracker red. Panels show typical snap shots of treated and untreated melanoma cells. (**b**) Cytotoxicity was evaluated by FACS analysis in melanoma cells following 4 h incubation with CTL. Melanoma cells either pretreated or not with 40 μM monensin for 2 h before conjugation were either unpulsed or pulsed with 10 μM antigenic peptide. Cytotoxicity is expressed as % of positive 7-AAD cells.(**c**) Efficient activation of CTL to lethal hit delivery after encountering melanoma cells treated or not with monensin. Target cells, either pretreated or not with 40 μM monensin were conjugated for 1 h with specific CTL. Cells were surface stained for CD107a. (**d**,**e**) Cytotoxicity was evaluated in melanoma cells following 4 h incubation with CTL. Melanoma cells were either untreated or treated with E64d or CI3 protease inhibitors. Melanoma cells were either unpulsed or peptide pulsed and cytotoxicity was quantified by measuring 7-AAD uptake by FACS analysis. (**d**) A representative experiment is shown; (**e**) pooled data from three independent experiments. Cytotoxicity is expressed as % of positive 7-AAD cells. (**f**) Perforin quanta (green) at the lytic synapse after 5 min conjugation with CTL of peptide-pulsed melanoma cells (either untreated or pretreated with 40 μM monensin). (**g**) Scheme depicting the fluorescence intensity quantification strategy for perforin deposits at the lytic synapse of melanoma cell/CTL conjugates. (**h**) Quantification of percentage of perforin fluorescence intensity on melanoma cell side of the lytic synapse in peptide-pulsed melanoma cells either pretreated or not with 40 μM monensin. Red bars indicate mean values of measured fluorescence. In all, 54 and 55 conjugates (for untreated and monensin-treated cells, respectively) formed by one melanoma cell and one CTL were scored. Data are from three independent experiments. In **b**,**c** and **e** results are expressed as mean±s.e.m. of three independent experiments. Scale bars, 5 μm. Unpaired Student's *t*-test using the GraphPad Prism software was used to determine the statistical significance of differences between the groups. ^***^P<0.001, ^**^P<0.01, ^NS^*P*>0.05.

Having observed that interfering with LLE trafficking in melanoma cells weakened their resistance to perforin-mediated cytotoxicity, we investigated whether alteration of melanoma cell lysosome function would also increase their sensitivity to CTL attack. In a first approach, we perturbed melanoma cell LLE function by using drugs altering the pH of those compartments. Melanoma cells were pretreated with 40 μM monensin, a carboxylic ionophore specific for monovalent cations that elevates vacuolar pH^36^. This treatment resulted in a profound alteration of melanoma cell LLE compartment as detected by impaired staining with the pH-dependent probe Lyso-Tracker Red (Fig. 7a). Monensin was thoroughly washed before co-culture with CTL. Monensin-treated melanoma cells exhibited enhanced cytotoxicity when compared with the untreated counterpart (Fig. 7b). Under the same experimental conditions, CTL activation was not affected, as detected by CD107a exposure on their surface (Fig. 7c).

Enhancement of CTL-mediated cytotoxicity following monensin treatment was confirmed in eight additional melanoma cell lines (Supplementary Fig. 9).

In further experiments, the impact of bafilomycin A1 and concanamycin A (two additional drugs altering acidic endosomal compartment pH^37^) was investigated. As shown in Supplementary Fig. 10, pre-treatment of melanoma cells with these drugs followed by washing before conjugation with CTL, significantly enhanced cytotoxicity.

In a second approach, we pretreated melanoma cells with inhibitors of proteolytic enzymes. Pretreatment of melanoma cells with E64d, a protease inhibitor targeting a limited number of protelytic enzymes including cathepsins (see Methods) or specific inhibitor of cathepsins (CI3), followed by extensive washing before conjugation with CTL, significantly increased cytotoxicity (Fig. 7d,e).

These results supported our finding that perforin can be degraded by melanoma cathepsins and further illustrated that the LLE pathway might serve as an important defence mechanism against CTL-mediated cytotoxicity in melanoma cells.

Finally, we investigated if it was possible to visualize, by confocal microscopy, synaptic quanta of perforin on melanoma cell surface. We stained therefore CTL/melanoma cell conjugates with anti-perforin antibodies followed by secondary antibodies labelled with QDots. This approach allowed us to detect synaptic perforin and to quantify the amount of early exocytosed perforin bound to the melanoma cell side of the synapse. We performed 3D confocal microscopy in conditions in which melanoma cells were either untreated or pretreated with monensin and conjugated for 5 min with CTL. As shown in Fig. 7f and Supplementary Movie 16, perforin staining was barely detectable on untreated melanoma cells. Conversely, synaptic perforin quanta were detected on the surface of melanoma cells that had been pretreated with monensin (Fig. 7f; Supplementary Movie 17). Measurement of perforin staining on a significant number of CTL/melanoma cell conjugates showed a significant increase in synaptic perforin staining in monensin-treated melanoma cells (Fig. 7g,h).

To further visualize this phenomenon, we performed 3D reconstruction of images in which CTL/melanoma cell conjugates were stained with antibodies directed against CD107a and perforin. The 3D images further show that in monensin-treated melanoma cells, perforin quanta decorate the synaptic side of the melanoma cell surface (Supplementary Movies 18,19,20).

Taken together, the above results illustrate that perforin, once released by CTL at the synaptic cleft, is rapidly destroyed by melanoma cells via a mechanism dependent on LLE proteolytic function.

## Discussion

In the present work, we investigated the dynamics of interaction between CTL and melanoma cells. We reveal a novel mechanism of melanoma cell defence from CTL attack, based on targeted trafficking of late LLE vesicles towards the synaptic area and on LLE burst exocytosis. This self-defence mechanism leads to synaptic degradation of perforin and failure in pore formation.

In this study, we took advantage of our previous observation that virus-specific CTLs are fully activated to lethal hit delivery when interacting with peptide-pulsed melanoma cells^22^. Using this cellular model we could therefore focus on melanoma cell mechanisms of resistance, with no concern for CTL activation. The fact that human CTL can be efficiently triggered to lethal hit delivery when interacting with peptide-pulsed melanoma cells (that might exhibit variable levels of HLA molecules) is not surprising. Others and we, indeed, put forth the notion that CTL are exquisitely sensitive to antigenic stimulation and exhibit saturating cytotoxicity responses when interacting with target cells displaying on their surface an extremely low number of specific peptide–MHC complexes^12^,^23^,^38^,^39^.

Our results shed light on the role played by lysosomal proteolytic enzymes in the regulation of CTL function. The interplay between cathepsins and various components of the CTL lytic cascade has been thoroughly investigated. It is well established that cathepsins contained within CTL lytic granules are instrumental for maturation of pro-caspases into caspases and for the efficient induction of target cell apoptosis^40^,^41^. Moreover, CTL granule CatB has been reported to restrain perforin function by degrading perforin bound on the CTL surface. This mechanism has been proposed to be instrumental in the protection of CTL from suicide or fratricide killing^31^. However, this function of CatB is controversial since it has been reported that in CatB knockout mice CTL can survive their own lytic granule secretion^32^.

While the functional role of cathepsins contained in lytic granules has been studied, whether and how proteolytic enzymes released by target cells at the lytic synapse might enhance or dampen the efficacy of lethal hit delivery remains to be elucidated. We provide here experimental support to the hypothesis that perforin can indeed be a substrate of cathepsins and that perforin proteolysis is instrumental for the survival of melanoma target cells.

Our results are in apparent contrast with previous data showing that purified perforin is a relatively poor substrate for CatB *in vitro*^32^. A possible explanation of this apparent discrepancy might be that several proteolytic enzymes contained in melanoma LLE and in lytic granules themselves might concur to perforin degradation. An alternative explanation is that cathepsins might activate additional proteolytic enzymes contained in melanoma cell lysosomes that would be responsible for perforin degradation.

An interesting question raised by our work concerns where perforin degradation actually occurs in CTL/melanoma cell conjugates. Our results are compatible with two scenarios. First, degradation might occur extracellularly on the melanoma cell side of the lytic synapse. Accordingly, melanoma cell lysosome secretion might allow synapse acidification thus creating a synaptic microenvironment facilitating hydrolases function. Such a scenario has been previously described for lysosomal exposure at the B-cell synapse for antigen extraction and processing^42^. A second scenario also compatible with our data is that perforin degradation might rapidly occur intracellularly on endocytosis.

We favour the hypothesis that perforin is degraded at the cell surface, since in our hands, perforin quanta at the lytic synapse are barely visible in untreated melanoma cells while they are detected on the cell surface following alteration of lysosomal pH (Fig. 7; Supplementary Movies 16–20).

Taken together, our results underline the role played by the endosomal compartment of target cells in regulating the process of perforin-mediated cytotoxicity. It has been reported that perforin triggers in target cells an early endosomal compartment-based reparation mechanism that regulates cell death by favouring the induction of apoptois as opposed to necrosis^43^,^44^. Here we extend this notion, by showing that, in CTL-resistant target cells, such as melanoma cells, the process of cell death is aborted by lysosomal proteases-mediated perforin degradation.

We also show that on interaction with cognate CTL, melanoma cell LLE vesicles are enriched at the lytic synapse. These results indicate that melanoma cells are capable of dedicated lysosomal polarization responses to face CTL lytic assault. The molecular mechanisms triggering such dedicated responses are presently elusive. We speculate that they might be triggered by localized calcium entry due to perforin-mediated pore formation at the synaptic area^45^.

An interesting aspect of our research is that we describe an important constitutive trafficking of lysosomal vesicles at the melanoma cell plasma membrane that is enhanced following contact with cognate CTL. Together, these results indicate that active lysosomal secretion might have been developed by melanoma cells as part of an immunoediting process occurring under the selective pressure of the immune system and might therefore serve to escape from CTL-mediated immune surveillance *in vivo*^46^.

It is tempting to speculate that LLE-mediated defence of target cells from CTL attack is not a prerogative of melanoma cells but is shared by other tumour cells. A previous study reported a correlation between the resistance to natural killer cell-mediated killing and reduced perforin staining in a human leukaemia cell line, however the molecular mechanisms of impaired perforin staining were not investigated^47^. Further research is required to define whether LLE secretion might be a general defence mechanism against the perforin/caspase pathway in cytotoxicity-resistant cells. A first indication in favour of this possibility comes from our results showing that conventional target cells expressing high levels of CD63 and CD107a are more resistant to CTL-mediated cytotoxicity than their CD63^low^CD107a^low^ counterpart (Supplementary Fig. 11).

A number of studies have demonstrated that anti-apoptotic pathways and other escape mechanisms, operate in cancer cells to mediate resistance to CTL or natural killer cell attack^20^,^21^,^48^,^49^,^50^. Our observation of an early defect in lethal hit delivery does not exclude that additional downstream pathways might operate in melanoma cells to generate resistance to CTL-mediated cytotoxicity. However, in our study, we show that the suppression of lysosome-based early defence mechanisms enhances cytotoxicity, indicating that this might be a relevant mechanism of defence among others.

It is well known that in melanoma patients the upregulation of PD-L1 in response to inflammatory mediators released by infiltrating T lymphocytes and by other cells of the microenvironment plays a crucial role in reducing ongoing immune responses^51^,^52^,^53^. It is tempting to speculate that LLE secretory burst might work as an early ‘innate' mechanism of defence at the beginning of immune cell attack to give time to melanoma cells to deploy late ‘adaptive' defence mechanisms against infiltrating lymphocytes, including PD-L1 upregulation. During late phases of the response, immune-checkpoint receptors would serve as key mechanisms of melanoma cell escape from immune surveillance. On patient treatment with antibodies directed against immune-checkpoint molecules, the LLE secretory burst might regain a primary role in melanoma cell defence from immune cell attack.

All in all, our results are compatible with a model in which LLE vesicle secretion at the lytic synapse by melanoma cells is a fundamental early molecular mechanism of cell resistance to CTL attack that acts during the first few minutes after the encounter with CTL and is *per se* sufficient to confer strong resistance to CTL attack. Nevertheless, this resistance mechanism is potentially complementary to other additional mechanisms of resistance that individual melanoma cells might develop, including upregulation of PD-L1 and resistance to apoptosis induction.

Various strategies are currently employed to potentiate CTL-mediated immune responses in melanoma patients with the goal of impairing tumour progressions. However, current clinical results are overall unsatisfactory^6^,^9^. Although melanoma vaccines using peptides emulsified in incomplete Freund's adjuvant, irradiated whole cells (either untreated or genetically modified to release immuno-stimulating factors), cell lysates or autologous dendritic cells carrying melanoma antigens are currently being assessed, clinical benefits have been obtained only on a small number of patients^6^,^9^. Therapies based on adoptive transfer of autologous *in vitro* expanded TILs are promising and can elicit clinical responses lasting for years in a fraction of treated patients, however these procedures are expensive and require lymphocyte depleting regimens that can expose patients to severe adverse effects^5^,^6^,^7^,^9^. Finally, therapies based on monoclonal antibodies targeting the CTLA-4/CD80-CD86 or the PD-1/PD-L1 axis are certainly very promising^6^,^9^,^54^, however they need to be optimized to establish the best compromise between clinical benefits and adverse effects.

Our results point out a new possible therapeutic path worth following to tackle melanoma resistance to immune surveillance from a new angle. Targeting lysosomal proteases-mediated defence of melanoma cells at the lytic synapse can be indeed complementary to any current immuno-stimulating strategy and can therefore provide a real clinical benefit. Protease inhibitors have been previously tested in the therapy of melanomas as well as of other tumours in an attempt to interfere with tumour invasion and metastasis^55^. It is tempting to speculate that the use of protease inhibitors might be re-considered to associate them to therapeutic strategies aiming at potentiating CTL responses.

In conclusion, our results underscore a previously unknown potential Achilles' heel of melanoma cells in respect to CTL-mediated cytotoxicity that might be exploited in clinical trials. They can inspire the pioneering of new therapeutic strategies to enhance CTL-mediated activity in melanoma patients by targeting the melanoma cell side of the immunological synapse.

## Methods

### Cell culture and transfection conditions

Human CD8^+^ T-cell lines were purified from healthy donor blood samples using the RosetteSep Human CD8^+^ T Cell Enrichment Cocktail (StemCell Technologies). For cloning, HLA-A2-restricted CD8^+^ T cells specific for the NLVPMVATV peptide or the VLAELVKQI peptide of the cytomegalovirus protein pp65 were single cell sorted into 96-U-bottom plates using a BD FACSAria II cell sorter using tetramer staining. Cells were cultured in RPMI 1640 medium supplemented with 8% human AB serum (PAA), minimum essential amino acids, HEPES and sodium pyruvate (Invitrogen), 100 IU ml^−1^ human rIL-2 and 50 ng ml^−1^ human rIL-15. CD8^+^ T-cell clones were stimulated in complete RPMI/HS medium containing 1 μg ml^−1^ PHA with 1 × 10^6^ per ml 35 Gy irradiated allogeneic peripheral blood mononuclear cells (isolated on Ficoll Paque Gradient from buffy coats of healthy donors) and 1 × 10^5^ per ml 70 Gy irradiated EBV-transformed B cells. Re-stimulation of clones was performed every 2 weeks. Blood samples were collected and processed following standard ethical procedures (Helsinki protocol), after obtaining written informed consent from each donor and approval for this study by the local ethical committee (Comité de Protection des Personnes Sud-Ouest et Outremer II).

The following HLA-A2^+^ cell lines were used as target cells: EBV-transformed B cells (JY^13^,^56^); HBL and D10 cells (isolated from metastatic melanoma patients, kindly provided by Dr G. Spagnoli, Basel, Switzerland); M17, M44 and M113 melanoma lines (isolated from metastatic melanoma patients, kindly provided by Dr F. Jotereau, Nantes, France), EB81-MEL.B, LB3110-MEL and LB2259-MEL.A (isolated from metastatic melanoma patients, kindly provided by Dr P. Coulie and N.V. Baren, Brussels, Belgium); ME275 (isolated from metastatic melanoma patients, kindly provided by Dr Daniel Speiser Ludwig Institute for Cancer Research Lausanne, Switzerland). The human acute T-cell leukaemia cell line Jurkat was from the ATCC collection. Jurkat cells are known to be sensitive to perforin and are therefore used for *in vitro* measurement of perforin lytic function^32^. Cells were authenticated on the basis of TCR/CD3 expression by FACS analysis. Our cell lines are routinely screened for mycoplasm contamination using the MycoAlert mycoplasma detection kit (Lonza).

Transfection of 1 × 10^6^ melanoma D10 cells with 4 μg plasmids coding for shRNA targeting SNAP-23 or a non-targeting shRNA plasmid (Sigma,SNAP-23: 5′-GTACCGGGAAACTCATTGACAGCTAAAGCTCGAGCTTTAGCTGTCAATGAGTTTCTTTTTTG-3′ and Control: no specific target) were performed using TransIT-X2 dynamic delivery system (Mirus), according to the manufacturer's recommendations.

The medium was replaced 8 h after transfection. Alternatively, 1 × 10^5^ melanoma D10 cells were transduced with 10 MOI lentiviral vector carrying the same plasmids over 6 h in RPMI-1640 containing 5% FCS and polybrene (4 μg ml^−1^). Transfected cells were selected by culturing them in the presence of 2 μg ml^−1^ puromycin for 7 days. Puromycin was added 48 h after transfection. Target cells were cultured in complete RPMI-1640 supplemented with 10% FCS. Transfection efficiency was evaluated by PCR and flow cytometry.

### FACS analysis

The following mAbs were used: Alexa Fluor 488 or Alexa Fluor 647 anti-human perforin antibody (5 μg ml^−1^, clone dG9; BioLegend), PE-Cy7 mouse anti-human CD107a (10 μg ml^−1^, cloneH4A3; BD Biosciences), Pacific Blue anti-human CD8 antibody (10 μg ml^−1^, clone RPA-T8; BioLegend) and FITC mouse anti-human CD63 (10 μg ml^−1^, cloneH5C6; BD Biosciences).

To distinguish CTL from target cells in the analysis, CTL were loaded with 1 μM CellTracker Green CMFDA (5-chloromethylfluorescein diacetate (Molecular Probes, Invitrogen) in RPMI for 15 min at 37 °C, prior conjugation with target cells. Alternatively, cells were not loaded with 0.1 μM CMFDA before conjugation instead CD8^+^ cells were identified by a staining with Pacific Blue anti-human CD8 antibody. Target cells were co-cultured with T cells in RPMI, 5% FCS/HEPES at two CTL versus one target cell ratio. At different time points, CTL/target cell co-culture cells were washed in ice-cold PBS containing 0.5 mM EDTA. Non-specific binding was prevented by 15 min incubation with 1% PBS containing 1% FCS which was used throughout the procedure as staining and washing buffer. Cells were stained with either specific antibodies or corresponding isotype controls. For cathepsin B expression on target cell surface cells were stained with a goat anti-human cathepsin B (10 μg ml^−1^, sc-6493; Santa Cruz) followed by an Alexa Fluor 647 donkey anti-goat Ab (10 μg ml^−1^, Invitrogen). Staining was performed on ice at 4 °C for 30 min. Samples were acquired using a FACS calibur or LSR II (Becton and Dickinson). Results were analysed using the FlowJo Pro software (Tree Star, Inc.).

### Cytotoxicity assay

Target cells were left unpulsed or pulsed with 10 μM antigenic peptide during 2 h at 37 °C/5% CO_2_, washed three times and subsequently transferred to a 96-well U-bottom plate at 25 × 10^3^ cells per 100 μl RPMI, 5% FCS/HEPES. CTL were previously stained with 0.1 μM CMFDA for 15 min at 37 °C/5% CO_2_, washed and added to the target cells at two CTL versus one target cell ratio (unless indicated) in 100 μl RPMI, 5% FCS/HEPES. Cells were pelleted for 1 min, 455*g* and incubated at 37 °C/5% CO_2_ for 4 h. In some experiments, target cells were pre-treated with either 40 μM monensin or 1 μM bafylomycin A1 or 1 μM concanamycin A and thoroughly washed before conjugation with CTL. In additional experiments, melanoma cells were pretreated for 16 h with 10 μM E64d (inhibitor of calpain, cathepsin B, H and L; Sigma) or 10 μM cathepsin inhibitor III (cathepsin B, H and L inhibitor; Merck-Millipore) in serum-free medium. During the last 2 h, cells were either unpulsed or pulsed with antigenic peptide. Cells were thoroughly washed before conjugation with CTL. Before FACS analysis, 0.25 μg 7-aminoactinomycin D (7-AAD; BD Biosciences) was added to each sample to measure the percentage of death targets. For the present study, a 4-h 7-ADD uptake cytotoxicity assay has the advantage, over other possible tests, used to measure melanoma cell survival and growth (such as colony formation assays), since it allows to measure cell death during a time window compatible with perforin-mediated cytotoxicity.

### Time-lapse microscopy monitoring of perforin pore formation

Target cells were pulsed with 10 μM peptide, washed and seeded at 1.5 × 10^5^ cells per well on poly-D-lysine-coated eight-well chambered slides (Ibidi, Munich, Germany) 5 min before imaging. Chambered slides were mounted on a heated stage within a temperature-controlled chamber maintained at 37 °C, and constant CO_2_ concentrations (5%). At the beginning of recording 2 × 10^5^ CTL, labelled with either 1 μM CMFDA or with 5μM TubulinTracker Green (Molecular Probes, loading was performed for 30 min at 37 °C/5% CO_2_) were added to chambered slides in the presence of 200 μM PI. Images of CTL/target cell conjugates were acquired for 1 h using either a Zeiss LSM 510 or a Zeiss LSM 710 microscope (Zoom factor × 63).

### Intracellular staining

Target cells were either unpulsed or pulsed with 10 μM antigenic peptide for 2 h at 37 °C in RPMI 5% FCS/HEPES and washed three times (in some experiments cells were pretreated with 40 μM monensin for 2 h). Conjugates of two CTL versus one target cell were then formed by 1 min centrifugation at 455*g*. On 5 or 15 min of co-incubation at 37 °C, cells were fixed with 3% paraformaldehyde, permeabilized with 0.1% saponin (in PBS/3% BSA/HEPES), and stained with the following primary antibodies: anti-human perforin mAb (10 μg ml^−1^, clone δG9; BD Pharmingen), anti-human CD63 mAb (10 μg ml^−1^, ab1318; Abcam), anti-human GrzB mAb (10 μg ml^−1^, clone G11; Santa Cruz Biotechnology), anti-human Serglycin mAb (10 μg ml^−1^, ab76512; Abcam) anti-human CD107a rabbit Ab (10 μg ml^−1^, ab24170; Abcam) and anti-human SNAP-23 (10 μg ml^−1^, Ab4114; Abcam). Primary Abs were followed by goat anti-mouse isotype-specific Ab or goat anti-rabbit Ab labelled with Alexa 488, Alexa 555, Alexa 647, Alexa 700 (10 μg ml^−1^) or goat anti-Mouse IgG conjugate (H+L) labelled with either QDot 525 or QDot 585 (0.02 μM, Molecular Probes). In some experiments, target cells are identified by Cell Tracker Blue (Life Technologies) staining. The samples were mounted in 90% glycerol-PBS containing 2.5% DABCO (Fluka) and examined using either a LSM 710 (Zeiss) or SP8 (Leica) confocal microscope over a × 63 Plan-Apochromat objective (1.4 oil). Electronic zoom 4 on LSM710 and 5 on SP8. Z-Stack images of optical sections were acquired throughout the cell volume. Alternatively, GrzB staining was evaluated by FACS analysis as described above.

### Image quantification

Images were scored by evaluating, for each experimental condition, at least 45 CTL/target cell conjugates in randomly selected fields from at least three independent experiments. GrzB staining was evaluated by scoring the percentage of positive target cells. To quantify the polarization of CD63^+^ vesicles towards the immunological synapse or synaptic enrichment of Av-SRho staining, unprocessed images were analysed by dividing each target cell into three equal regions. Fluorescence was measured using the ROI statistics tool of the ImageJ software on image projections in two equally defined volumes (one at the synaptic area and the other at the distal area). To exclude CD63 or Av-SRho staining of CTL, a region was carefully drawn on the melanoma cell side of the lytic synapse. Results were reported as the fold increase of the corresponding staining integrated FI at the synaptic region divided by the same FI measured at the distal region (see schemes drawn in Fig. 3). For quantification of perforin on melanoma cells, the lytic synapse was divided into two equal volumes, one on the CTL and one on the melanoma cell side. Total perforin FI at the lytic synapse of both volumes was acquired on image projections of CTL/melanoma conjugates. Perforin on melanoma cells was quantified as the percentage of perforin FI at the defined melanoma cell volume (red box) divided by the totality of perforin FI (black box) at the lytic synapse (% of perforin FI=(perforin FI on melanoma cell/total perforin FI) × 100; see schemes drawn in Fig. 7) using the ImageJ software. The above-described measurements were confirmed by analysing FI in melanoma cells z-stack projections using the Region Measurements of the Metamorph software (Universal Imaging). In further experiments z-stack images of CTL interacting with melanoma cells were reconstructed and analysed using the Surface tool of the Imaris software.

### Monitoring melanoma cell LLE trafficking and secretion

To quantify lysosomal localization at the lytic synapse, wild-type D10 cells or SNAP-23 silenced D10 cells were infected with 40 particles per cell of baculovirus coding for CD107a-GFP (Cell Light Lysosomes; Invitrogen) overnight. Cells were either unpulsed or pulsed with 10 μM peptide and monitored during interaction with CMFDA or Av-SRho loaded CTL. Time-lapse video microscopy was performed for 1 h using a Nikon inverted spinning disk confocal microscope equipped with a back-thinned charge-coupled device camera (Evolve; Photometrics, Tucson, AZ, 512 × 512 pixels), equipped with a temperature-controlled chamber maintained at 37 °C, and constant CO_2_ concentration (5%), allowing acquisition of multi-position images using an NA1.3/ × 40 oil-immersion objective piloted by the Metamorph7 software. The images were processed using ImageJ. CD107a-GFP-integrated FI at the lytic synapse was quantified for several conjugates and reported as fold increase over the initial intensity at time 0 of conjugate formation.

To measure the exposure of the lysosomal compartments of melanoma cells, peptide-pulsed wild-type D10 or SNAP-23 silenced D10 cells were seeded into poly-D-lysine-coated chambered slides in 100 μl medium containing 8 μg ml^−1^ Av-SRho. Av-SRho (red) uptake in the presence or absence of CMFDA or Avidin-Alexa488-loaded CTL was monitored for 1 h using a Nikon inverted spinning disk confocal microscope.

### Measurement of perforin lytic activity

Human purified perforin (Enzo Life) permabilizing activity was determined by 1 h incubation of 1 × 10^6^ Jurkat cells with 125 ng of perforin following manufacturer's recommended conditions. Perforin was pretreated or not for 2 h at 37 °C with 500 ng ml^−1^ purified human liver cathepsin B (CatB, Merck-Millipore), or CatB that was previously co-incubated with 10 μM CA074 (Merck-Millipore) for 30 min at 37 °C. Cell permeabilization was measured in the presence of 25 μg ml^−1^ PI by FACS analysis. In some experiments, lytic granule lysates were pretreated or not with melanoma cell vesicular fraction lysates for 2 h at 37 °C. Melanoma cell vesicular fraction lysates were previously co-incubated or not with 10 μM CA074 (Merck-Millipore) for 30 min at 37 °C before being incubated with lytic granule lysates. Intracellular vesicles isolation from melanoma cells was performed following a published protocol^57^, that we previously employed for lytic granule isolation with minor modifications (see below).

### Western blot analysis of perforin cleavage

Granule isolation was performed following a published protocol^57^ with minor modifications. Briefly, CD8^+^ T cells were washed three times with ice-cold PBS and reconstituted in 2 × 10^7^cells per ml of relaxation buffer: 130 mM KCl; 5 mM NaCl; 2 mM MgCl_2_; 1 mM disodium-ATP/10 mM HEPES pH 7.4. Cells were disrupted by N_2_ cavitation at 400 p.s.i. and the cell suspension was collected in the presence of 4 mM EGTA. The cell lysate was separated into a post-nuclear supernatant (SN1) and a pellet (P1) by centrifugation at 800*g* for 10 min at 4 °C. SN1 was submitted to another centrifugation step at 20,000*g* for 30 min at 4 °C, which resulted in a cytosolic supernatant (SN2) and a total vesicular extract enriched in the pellet P2. For melanoma cell lysates, 5 × 10^6^ cells were diluted in cytobuster for protein extract following manufacture protocol (Merck-Millipore). The supernatant was collected by centrifugation. Aliquots of 500 ng of total protein from lytic granule extract lysates were either treated with 500 ng ml^−1^ CatB or an increasing concentration of melanoma cell lysate (0.5, 1and 3 μg ml^−1^ total protein) for 2 h at 37 °C, then mixed with SDS sample buffer. Reduced 10% SDS gels were run and blotted onto a nitrocellulose membrane. After blocking with 3% nonfat dry milk in Tris-buffered saline with Tween (TBST) for 1 h, the membranes were incubated with 10 μg ml^−1^ anti-perforin (clone H-315; Santa Cruz Biotechnology) mAb overnight at 4 °C, followed by 2 h of incubation with an HRP-anti-rabbit IgG (SouthernBiotech). The blots were developed using ECL (GE Healthcare). Western blot images were acquired using a ChemiDoc MP System (Bio-Rad). In some experiments, CatB was pretreated with 10 μM CA074 for 30 min at 37 °C before addition of the mixture on lytic granule lysates. Band intensity was quantified using ImageJ software as raw intensity over non-treated lytic granule lysate. Uncropped western blots are presented in Supplementary Fig. 7.

### cDNA synthesis and PCR

RNA isolation was performed using the RNeasy Mini Kit (Roche Life Sciences), and RNA concentration was assessed using the NanoDrop 1000 (Thermo Scientific) system RNA was converted to complementary DNA (cDNA) using the Applied Biosystems High Capacity cDNA Reverse Transcription Kit (Life Technologies).

The gene expression of SNAP-23 (Hs001870775_m1; context sequence: 5′-TCACAGACAAGGCTGACACCAACAG-3′) was evaluated by real-time quantitative PCR (RT qPCR) using TaqMan gene expression assays (Applied Biosystems) according to the indicated protocol, using a LightCycler 480 System (Roche Life Sciences). All reactions were performed in triplicates and relative gene expression levels were evaluated using the comparative CT (threshold cycle) method (2-deltaCT). GAPDH (Hs03929097_g1; context sequence: 5′-CAAGAGGAAGAGAGAGACCCTCACT-3′) was used as endogenous controls for normalization.

### Statistical analysis

Unpaired Student's *t*-test using the GraphPad Prism software (version 6; GraphPad) was used to determine the statistical significance of differences between the groups.

## Additional information

**How to cite this article:** Khazen, R. *et al*. Melanoma cell lysosome secretory burst neutralizes the CTL-mediated cytotoxicity at the lytic synapse. *Nat. Commun.* 7:10823 doi: 10.1038/ncomms10823 (2016).

## Supplementary Material

Supplementary Information Supplementary Figures 1-11

Supplementary Movie 1 The video shows the interaction between a peptide pulsed JY cell and tubulin tracker green loaded CTL for 1 hour in the presence of PI (red). Cells were inspected using a confocal laser-scanning microscope (LSM 510; zoom factor 63X) to monitor PI entry. Recording time is indicated in minutes in upper left corner.

Supplementary Movie 2 The video shows the interaction between a peptide pulsed D10 cell and tubulin tracker green loaded CTL for 1 hour in the presence of PI (red). Cells were inspected using a confocal laser-scanning microscope (LSM 510; zoom factor 63X) to monitor PI entry. Recording time is indicated in minutes in upper left corner.

Supplementary Movie 3 JY cells were seeded in chambered wells in the presence of 8µg ml-1 Av-SRho (pseudo color) and monitored for 2 hours using a confocal spinning disk microscope to visualize constitutive vesicular exocytosis. A typical cell is shown; data are representative of three independent experiments.

Supplementary Movie 4 D10 cells were seeded in chambered wells in the presence of 8µg ml-1 AvSRho (pseudo color) and monitored for 2 hours using a confocal spinning disk microscope to visualize constitutive vesicular exocytosis. Three typical cells are shown; data are representative of three independent experiments.

Supplementary Movie 5 The video shows interaction between a peptide pulsed D10 cell expressing CD107a-GFP (pseudo color) and two CTL (previously loaded with Av-SRho, red). Cells 5 were inspected using confocal spinning disk microscope. Recording time is indicated in minutes in upper left corner.

Supplementary Movie 6 The video shows interaction between a peptide pulsed D10 cell interacting with a CTL (previously loaded with CMFDA, green) in the presence of 8µg ml-1 Av-SRho (pseudo color). Cells were inspected using confocal spinning disk microscope. Recording time is indicated in minutes in upper left corner.

Supplementary Movie 7 The video shows interaction between unpulsed D10 cells interacting with CTL (previously loaded with CMFDA, green) in the presence of 8µg ml-1 Av-SRho (pseudo color). Cells were inspected using confocal spinning disk microscope. Recording time is indicated in minutes in upper left corner.

Supplementary Movie 8 The video shows a peptide pulsed D10 cell interacting with a CTL (that was previously loaded with Av-Alexa488, green) in the presence of 8µg ml-1 Av-SRho (red). Cells were inspected using a confocal spinning disk microscope. Recording time is indicated in minutes in upper left corner.

Supplementary Movie 9 The video shows interactions between a peptide pulsed JY cell and two CTL (previously loaded with CMFDA, green) in the presence of 8µg ml-1 Av-SRho (pseudo color). Cells were inspected using confocal spinning disk microscope. Recording time is indicated in minutes in upper left corner.

Supplementary Movie 10 The video shows interactions between a peptide pulsed JY cell and a CTL (previously loaded with CMFDA, green) in the presence of 8µg ml-1 Av-SRho (pseudo color). Cells were inspected using confocal spinning disk microscope. Recording time is indicated in minutes in upper left corner.

Supplementary Movie 11 The video shows interactions between SNAP-23 silenced peptide-pulsed D10 cells expressing CD107a-GFP (pseudo color) and a CTL (previously loaded with AvSRho, red). Cells were inspected using confocal spinning disk microscope. Recording time is indicated in minutes in upper left corner

Supplementary Movie 12 The video shows interactions between SNAP-23 silenced peptide-pulsed D10 cells interacting with CTL (previously loaded with CMFDA, green) in the presence of 8µg/ml Av-SRho (pseudo color). Cells were inspected using confocal spinning disk microscope. Recording time is indicated in minutes in upper left corner.

Supplementary Movie 13 The video shows interactions between control shRNA transfected peptidepulsed D10 cells expressing CD107a-GFP (pseudo color) and a CTL (previously loaded with Av-SRho, red). Cells were inspected using confocal spinning disk microscope. Recording time is indicated in minutes in upper left corner.

Supplementary Movie 14 The video shows interactions between control shRNA transfected peptidepulsed D10 cells interacting with CTL (previously loaded with CMFDA, green) in the presence of 8µg ml-1 Av-SRho (pseudo color). Cells were inspected using confocal spinning disk microscope. Recording time is indicated in minutes in upper left corner.

Supplementary Movie 15 The video shows interactions between control shRNA transfected peptidepulsed D10 interacting with CTL (previously loaded with Av-SRho, red) in the presence of 8µg ml-1 Av-Alexa488 (pseudo color). Cells were inspected using confocal spinning disk microscope. Recording time is indicated in minutes in upper left corner

Supplementary Movie 16 The movie shows a 3-D reconstruction of a conjugate formed between a CTL and an untreated melanoma cell. Cells were conjugated for 5 minutes and stained for perforin (green).

Supplementary Movie 17 The movie shows a 3-D reconstruction of a conjugate formed between a CTL and a melanoma cell that was pretreated with monensin. Cells were conjugated for 5 minutes and stained for perforin (green).

Supplementary Movie 18 The movie shows a 3-D reconstruction of a conjugate formed between a CTL and an untreated melanoma cell. Cells were conjugated for 5 minutes and stained for perforin and CD107a (blue). The 3-D reconstructed images were processed using the Imaris software to show in green the perforin co-localizing with CD107a (mostly found intracellularly in CTL only), and in red the perforin not colocalizing with CD107a (undetectable in this movie).

Supplementary Movie 19 The movie shows a 3-D reconstruction of a conjugate formed between a CTL and a melanoma cell that was pretreated with monensin. Cells were conjugated for 5 minutes and stained for perforin and CD107a (blue). The 3-D reconstructed images were 8 processed using the Imaris software to show in green the perforin co-localizing with CD107a (mostly found intracellularly in CTL only), and in red the perforin not colocalizing with CD107a (detected extracellularly).

Supplementary Movie 20 The movie shows a 3-D reconstruction of a conjugate formed between a CTL and a melanoma cell that was pretreated with monensin. Cells were conjugated for 5 minutes and stained for perforin and CD107a (blue). The 3-D reconstructed images were processed using the Imaris software to show in green the perforin co-localizing with CD107a (mostly found intracellularly in CTL only), and in red the perforin not colocalizing with CD107a (detected extracellularly).

## References

[b1] Griffiths G. M., Tsun A. & Stinchcombe J. C. The immunological synapse: a focal point for endocytosis and exocytosis. J. Cell Biol. 189, 397–406 (2010). 20439993 10.1083/jcb.201002027 PMC2867296

[b2] Huse M., Quann E. J. & Davis M. M. Shouts, whispers and the kiss of death: directional secretion in T cells. Nat. Immunol. 9, 1105–1111 (2008). 18800163 10.1038/ni.f.215 PMC2905669

[b3] Chen D. S. & Mellman I. Oncology meets immunology: the cancer-immunity cycle. Immunity 39, 1–10 (2013). 23890059 10.1016/j.immuni.2013.07.012

[b4] Coulie P. G., Van den Eynde B. J., van der Bruggen P. & Boon T. Tumour antigens recognized by T lymphocytes: at the core of cancer immunotherapy. Nat. Rev. Cancer 14, 135–146 (2014). 24457417 10.1038/nrc3670

[b5] Rosenberg S. A. & Restifo N. P. Adoptive cell transfer as personalized immunotherapy for human cancer. Science 348, 62–68 (2015). 25838374 10.1126/science.aaa4967 PMC6295668

[b6] Gajewski T. F. . Cancer immunotherapy strategies based on overcoming barriers within the tumor microenvironment. Curr. Opin. Immunol. 25, 268–276 (2013). 23579075 10.1016/j.coi.2013.02.009

[b7] Kalos M. & June C. H. Adoptive T cell transfer for cancer immunotherapy in the era of synthetic biology. Immunity 39, 49–60 (2013). 23890063 10.1016/j.immuni.2013.07.002 PMC3809038

[b8] Wimmers F., Schreibelt G., Skold A. E., Figdor C. G. & De Vries I. J. Paradigm shift in dendritic cell-based immunotherapy: from *in vitro* generated monocyte-derived DCs to naturally circulating DC subsets. Front. Immunol. 5, 165 (2014). 24782868 10.3389/fimmu.2014.00165 PMC3990057

[b9] Aris M. & Barrio M. M. Combining immunotherapy with oncogene-targeted therapy: a new road for melanoma treatment. Front. Immunol. 6, 46 (2015). 25709607 10.3389/fimmu.2015.00046 PMC4321613

[b10] Stinchcombe J. C., Bossi G., Booth S. & Griffiths G. M. The immunological synapse of CTL contains a secretory domain and membrane bridges. Immunity 15, 751–761 (2001). 11728337 10.1016/s1074-7613(01)00234-5

[b11] Law R. H. P. . The structural basis for membrane binding and pore formation by lymphocyte perforin. Nature 468, 447–U277 (2010). 21037563 10.1038/nature09518

[b12] Faroudi M., Zaru R., Paulet P., Muller S. & Valitutti S. Cutting edge: T lymphocyte activation by repeated immunological synapse formation and intermittent signaling. J. Immunol. 171, 1128–1132 (2003). 12874197 10.4049/jimmunol.171.3.1128

[b13] Bertrand F. . An initial and rapid step of lytic granule secretion precedes microtubule organizing center polarization at the cytotoxic T lymphocyte/target cell synapse. Proc. Natl Acad. Sci. USA 110, 6073–6078 (2013). 23536289 10.1073/pnas.1218640110 PMC3625254

[b14] Lopez J. A., Brennan A. J., Whisstock J. C., Voskoboinik I. & Trapani J. A. Protecting a serial killer: pathways for perforin trafficking and self-defence ensure sequential target cell death. Trends Immunol. 33, 406–412 (2012). 22608996 10.1016/j.it.2012.04.001

[b15] Baran K. . The molecular basis for perforin oligomerization and transmembrane pore assembly. Immunity 30, 684–695 (2009). 19446473 10.1016/j.immuni.2009.03.016

[b16] Lopez J. A. . Perforin forms transient pores on the target cell plasma membrane to facilitate rapid access of granzymes during killer cell attack. Blood 121, 2659–2668 (2013). 23377437 10.1182/blood-2012-07-446146

[b17] Thiery J. . Perforin pores in the endosomal membrane trigger the release of endocytosed granzyme B into the cytosol of target cells. Nat. Immunol. 12, 770–U146 (2011). 21685908 10.1038/ni.2050 PMC3140544

[b18] de Saint Basile G., Menasche G. & Fischer A. Molecular mechanisms of biogenesis and exocytosis of cytotoxic granules. Nat. Rev. Immunol. 10, 568–579 (2010). 20634814 10.1038/nri2803

[b19] Voskoboinik I. & Trapani J. A. Perforinopathy: a spectrum of human immune disease caused by defective perforin delivery or function. Front. Immunol. 4, 441 (2013). 24376445 10.3389/fimmu.2013.00441 PMC3860100

[b20] Zhuang L. . Mcl-1, Bcl-XL and Stat3 expression are associated with progression of melanoma whereas Bcl-2, AP-2 and MITF levels decrease during progression of melanoma. Mod. Pathol. 20, 416–426 (2007). 17384650 10.1038/modpathol.3800750

[b21] Lickliter J. D. . Small-molecule Bcl-2 inhibitors sensitise tumour cells to immune-mediated destruction. Br. J. Cancer. 96, 600–608 (2007). 17311012 10.1038/sj.bjc.6603599 PMC2360057

[b22] Caramalho I., Faroudi M., Padovan E., Muller S. & Valitutti S. Visualizing CTL/melanoma cell interactions: multiple hits must be delivered for tumour cell annihilation. J. Cell. Mol. Med. 13, 3834–3846 (2009). 19017355 10.1111/j.1582-4934.2008.00586.x PMC4516531

[b23] Valitutti S., Muller S., Dessing M. & Lanzavecchia A. Different responses are elicited in cytotoxic T lymphocytes by different levels of T cell receptor occupancy. J. Exp. Med. 183, 1917–1921 (1996). 8666949 10.1084/jem.183.4.1917 PMC2192499

[b24] Zimmerer R. M. . Functional features of cancer stem cells in melanoma cell lines. Cancer Cell. Int. 13, 78 (2013). 23915418 10.1186/1475-2867-13-78 PMC3765139

[b25] Lopez J. A. . Rapid and unidirectional perforin pore delivery at the cytotoxic immune synapse. J. Immunol. 191, 2328–2334 (2013). 23885110 10.4049/jimmunol.1301205

[b26] Reddy A., Caler E. V. & Andrews N. W. Plasma membrane repair is mediated by Ca(2+)-regulated exocytosis of lysosomes. Cell 106, 157–169 (2001). 11511344 10.1016/s0092-8674(01)00421-4

[b27] Jimenez A. J. . ESCRT machinery is required for plasma membrane repair. Science 343, 1247136 (2014). 24482116 10.1126/science.1247136

[b28] Joulia R. . Mast cells form antibody-dependent degranulatory synapse for dedicated secretion and defence. Nat. Commun. 6, 6174 (2015). 25629393 10.1038/ncomms7174

[b29] Korpetinou A. . Serglycin: at the crossroad of inflammation and malignancy. Front. Oncol. 3, 327 (2014). 24455486 10.3389/fonc.2013.00327 PMC3888995

[b30] Meen A. J. . Serglycin is a major proteoglycan in polarized human endothelial cells and is implicated in the secretion of the chemokine GRO alpha/CXCL1. J. Biol. Chem. 286, 2636–2647 (2011). 21075844 10.1074/jbc.M110.151944 PMC3024759

[b31] Balaji K. N., Schaschke N., Machleidt W., Catalfamo M. & Henkart P. A. Surface cathepsin B protects cytotoxic lymphocytes from self-destruction after degranulation. J. Exp. Med. 196, 493–503 (2002). 12186841 10.1084/jem.20011836 PMC2196055

[b32] Baran K. . Cytotoxic T lymphocytes from cathepsin B-deficient mice survive normally *in vitro* and *in vivo* after encountering and killing target cells. J. Biol. Chem. 281, 30485–30491 (2006). 16914553 10.1074/jbc.M602007200

[b33] Matarrese P. . Cathepsin B inhibition interferes with metastatic potential of human melanoma: an *in vitro* and *in vivo* study. Mol. Cancer. 9, 207 (2010). 20684763 10.1186/1476-4598-9-207 PMC2925371

[b34] Rao S. K., Huynh C., Proux-Gillardeaux V., Galli T. & Andrews N. W. Identification of SNAREs involved in synaptotagmin VII-regulated lysosomal exocytosis. J. Biol. Chem. 279, 20471–20479 (2004). 14993220 10.1074/jbc.M400798200

[b35] Soares H. . Regulated vesicle fusion generates signaling nanoterritories that control T cell activation at the immunological synapse. J. Exp. Med. 210, 2415–2433 (2013). 24101378 10.1084/jem.20130150 PMC3804939

[b36] Misinzo G., Delputte P. L. & Nauwynck H. J. Inhibition of endosome-lysosome system acidification enhances porcine circovirus 2 infection of porcine epithelial cells. J. Virol. 82, 1128–1135 (2008). 18032516 10.1128/JVI.01229-07 PMC2224462

[b37] Valitutti S., Muller S., Salio M. & Lanzavecchia A. Degradation of T cell receptor (TCR)-CD3-xi complexes after antigenic stimulation. J. Exp. Med. 185, 1859–1864 (1997). 9151711 10.1084/jem.185.10.1859 PMC2196323

[b38] Sykulev Y., Joo M., Vturina I., Tsomides T. J. & Eisen H. N. Evidence that a single peptide-MHC complex on a target cell can elicit a cytolytic T cell response. Immunity 4, 565–571 (1996). 8673703 10.1016/s1074-7613(00)80483-5

[b39] Purbhoo M. A., Irvine D. J., Huppa J. B. & Davis M. M. T cell killing does not require the formation of a stable mature immunological synapse. Nat. Immunol. 5, 524–530 (2004). 15048111 10.1038/ni1058

[b40] Pham C. T. N. & Ley T. J. Dipeptidyl peptidase I is required for the processing and activation of granzymes A and B *in vivo*. Proc. Natl Acad. Sci. USA 96, 8627–8632 (1999). 10411926 10.1073/pnas.96.15.8627 PMC17567

[b41] D'Angelo M. E. . Cathepsin H is an additional convertase of pro-granzyme B. J. Biol. Chem. 285, 20514–20519 (2010). 20435891 10.1074/jbc.M109.094573 PMC2898313

[b42] Yuseff M. I. . Polarized secretion of lysosomes at the B cell synapse couples antigen extraction to processing and presentation. Immunity 35, 361–374 (2011). 21820334 10.1016/j.immuni.2011.07.008

[b43] Keefe D. . Perforin triggers a plasma membrane-repair response that facilitates CTL induction of apoptosis. Immunity 23, 249–262 (2005). 16169498 10.1016/j.immuni.2005.08.001

[b44] Thiery J. . Perforin activates clathrin- and dynamin-dependent endocytosis, which is required for plasma membrane repair and delivery of granzyme B for granzyme-mediated apoptosis. Blood 115, 1582–1593 (2010). 20038786 10.1182/blood-2009-10-246116 PMC2830763

[b45] Reddy A., Caler E. & Andrews N. W. Plasma membrane repair is mediated by Ca2+-regulated exocytosis of lysosomes. Mol. Biol. Cell 12, 266A–266A (2001). 10.1016/s0092-8674(01)00421-4 11511344

[b46] Schreiber R. D., Old L. J. & Smyth M. J. Cancer immunoediting: integrating immunity's roles in cancer suppression and promotion. Science 331, 1565–1570 (2011). 21436444 10.1126/science.1203486

[b47] Lehmann C., Zeis M., Schmitz N. & Uharek L. Impaired binding of perforin on the surface of tumor cells is a cause of target cell resistance against cytotoxic effector cells. Blood 96, 594–600 (2000). 10887123

[b48] Jazirehi A. R., Nazarian R., Torres-Collado A. X. & Economou J. S. Aberrant apoptotic machinery confers melanoma dual resistance to BRAF(V600E) inhibitor and immune effector cells: immunosensitization by a histone deacetylase inhibitor. Am. J. Clin. Exp. Immunol. 3, 43–56 (2014). 24660121 PMC3960761

[b49] Jazirehi A. R., Baritaki S., Koya R. C., Bonavida B. & Economou J. S. Molecular mechanism of MART-1+/A*0201+ human melanoma resistance to specific CTL-killing despite functional tumor-CTL interaction. Cancer Res. 71, 1406–1417 (2011). 21159666 10.1158/0008-5472.CAN-10-1296 PMC4180868

[b50] Murakami T. . Immune evasion by murine melanoma mediated through CC chemokine receptor-10. J. Exp. Med. 198, 1337–1347 (2003). 14581607 10.1084/jem.20030593 PMC2194242

[b51] Dong H. . Tumor-associated B7-H1 promotes T-cell apoptosis: a potential mechanism of immune evasion. Nat. Med. 8, 793–800 (2002). 12091876 10.1038/nm730

[b52] Taube J. M. Emerging immunologic biomarkers: setting the (TNM-immune) stage. Clin. Cancer Res. 20, 2023–2025 (2014). 24634379 10.1158/1078-0432.CCR-14-0328 PMC4036638

[b53] Ribas A. Clinical development of the anti-CTLA-4 antibody tremelimumab. Semin. Oncol. 37, 450–454 (2010). 21074059 10.1053/j.seminoncol.2010.09.010

[b54] Lindsay C. R., Spiliopoulou P. & Waterston A. Blinded by the light: why the treatment of metastatic melanoma has created a new paradigm for the management of cancer. Ther. Adv. Med. Oncol. 7, 107–121 (2015). 25755683 10.1177/1758834014566619 PMC4346213

[b55] Frohlich E. Proteases in cutaneous malignant melanoma: relevance as biomarker and therapeutic target. Cell Mol. Life Sci. 67, 3947–3960 (2010). 20686912 10.1007/s00018-010-0469-5 PMC11115755

[b56] Vasconcelos Z. . Individual human cytotoxic T lymphocytes exhibit intraclonal heterogeneity during sustained killing. Cell Rep. 11, 1474–1485 (2015). 26027932 10.1016/j.celrep.2015.05.002

[b57] Sanchez-Ruiz Y., Valitutti S. & Dupre L. Stepwise maturation of lytic granules during differentiation and activation of human CD8+ T lymphocytes. PLoS ONE 6, e27057 (2011). 22073254 10.1371/journal.pone.0027057 PMC3208563

