# Supplementary material for: Melanoma cell lysosome secretory burst neutralizes the CTL-mediated cytotoxicity at the lytic synapse
Source: Nat Commun. 2016 Mar 4;7:10823. doi: 10.1038/ncomms10823 (PMC4785227; doi:10.1038/ncomms10823)
Supplement: Supplementary Information — Supplementary Figures 1-11 [file ncomms10823-s1.pdf]

Supplementary Figure-1

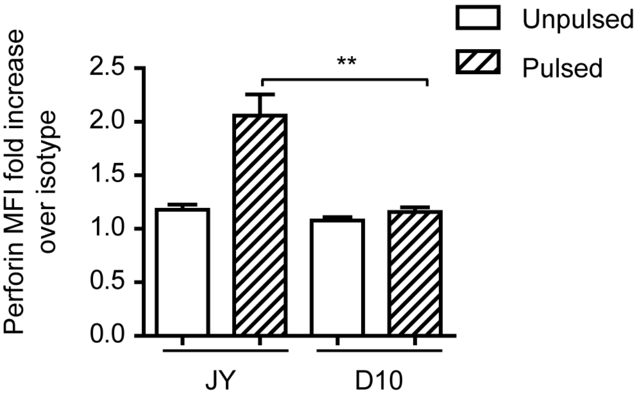

Supplementary Figure 1. **Limited perforin staining on melanoma cells.** Perforin staining on the surface of conventional target cells (JY) and melanoma cells (D10) following conjugation with CTL. Target cells were either unpulsed or pulsed with10μm antigenic peptide. The fold increase of median fluorescence intensity of the entire cell population over the isotype control is shown. Results are expressed as mean ± SEM of 6 independent experiments. Unpaired Student's t-test using the GraphPad Prism software (version 6; GraphPad) was used to determine the statistical significance of differences between the groups. **P<0.01.

Supplementary Figure-2

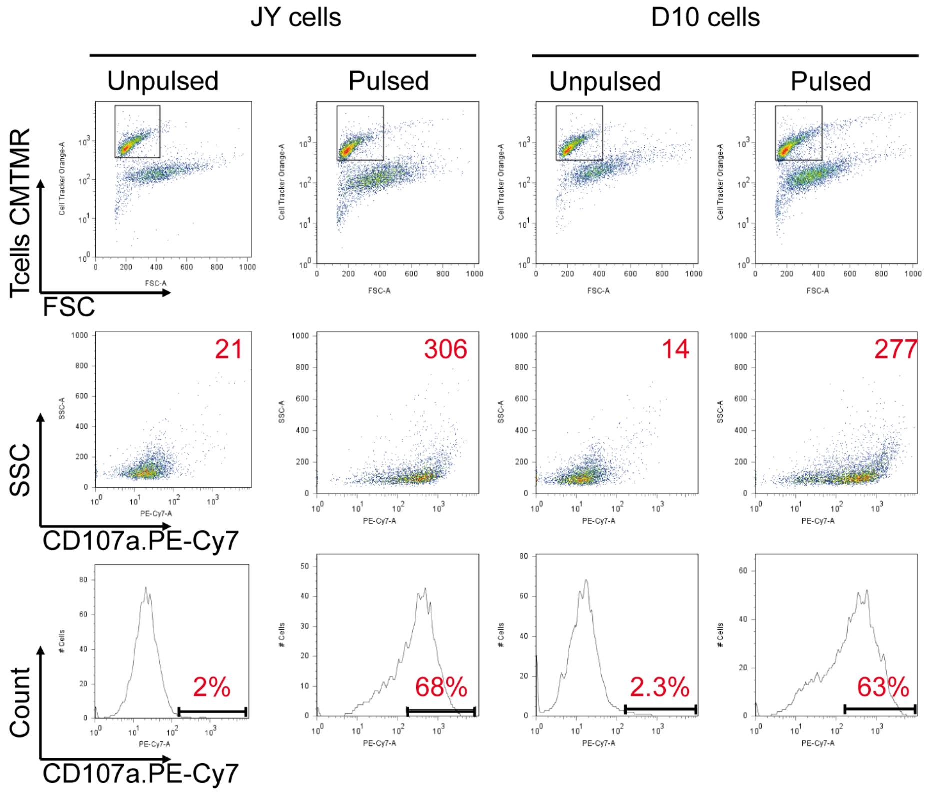

Supplementary Figure 2. **Expression of CD107a on the surface of CTL following interaction with melanoma cells or with conventional target cells.** The gating strategy and typical results of CD107a up-regulation on the surface of CTL interacting for 1 hour with either JY cells or with D10 cells either unpulsed or pulsed with 10µM peptide concentration is shown. Data are from one representative experiment out of three.

Supplementary Figure-3

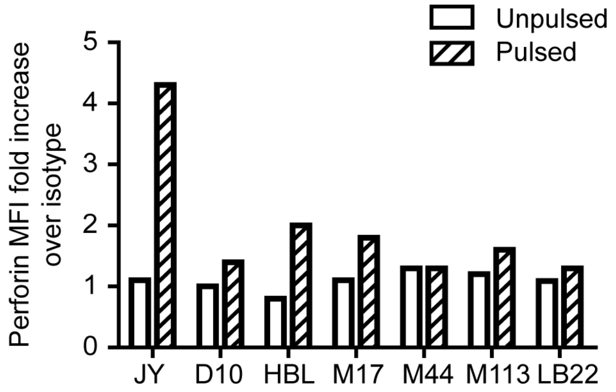

Supplementary Figure 3. **Perforin staining on several melanoma cell lines.** D10, HBL, M17, M44, M113, LB22 and JY cells were either unpulsed or pulsed with 10µM antigenic peptide. Cells were conjugated with CTL for 1 hour. Cells were assessed for perforin staining on their surface and analyzed by FACS. Data are from one representative experiment out of two.

Supplementary Figure-4

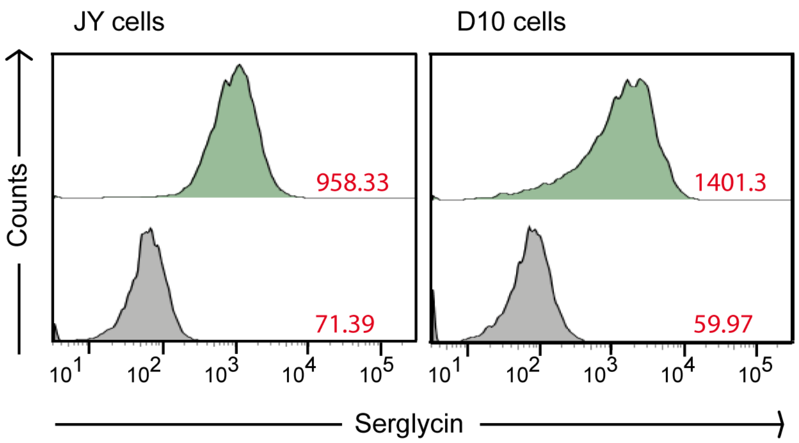

Supplementary Figure 4. **Serglycin expression in target cells.** Typical plots of intracellular staining for serglycin in D10 and JY cells are shown. Results are from one representative experiment out of two performed in triplicates.

Supplementary Figure-5

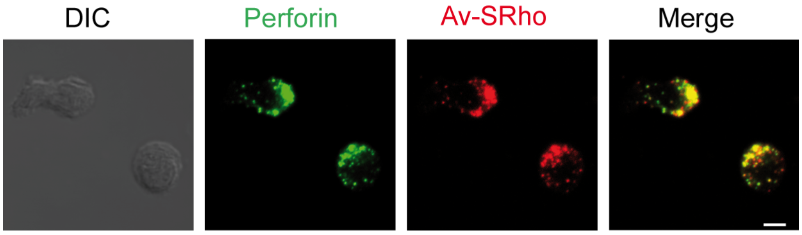

Supplementary Figure 5. **Av-SRho staining in CTL can be used as a marker of lytic granules.** CTL were cultured in the presence of 8µg/ml of Av-SRho (red) overnight. Cells were fixed, permeabilized and stained for perforin (green).

Supplementary Figure-6

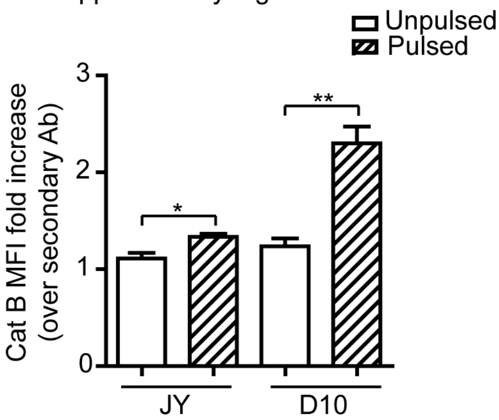

Supplementary Figure 6. **Cathepsin B exposure on the surface of target cells upon interaction with CTL.** JY cells and D10 cells either unpulsed or pulsed with 10µM peptide were conjugated for 15 minutes with CTL and stained for surface expression of cathepsisn B. The fold increase of median fluorescence intensity of the entire cell population over the secondary antibody is shown. Results are expressed as mean ± SEM of 3 independent experiments. Unpaired Student's t-test using the GraphPad Prism software (version 6; GraphPad) was used to determine the statistical significance of differences between the groups. **P<0.01; *P<0.05.

Supplementary Figure-7

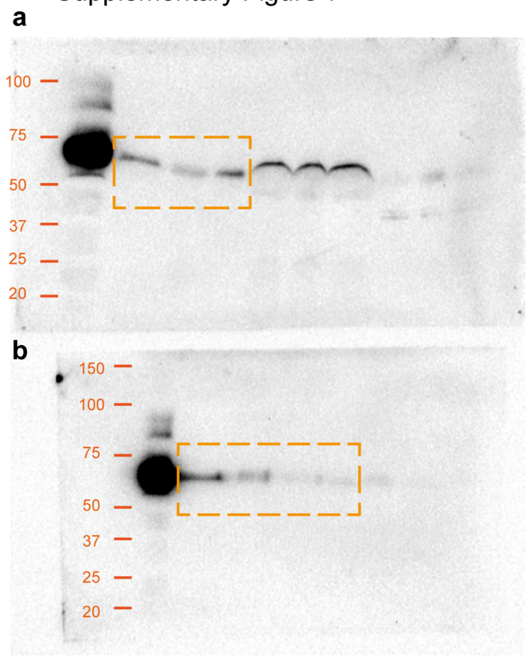

Supplementary Figure 7. **Uncropped Western Blots related to Figure 5.** The original Western Blots for results shown in Figure 5c and 5d are shown in panel a and b respectively.

Supplementary Figure-8

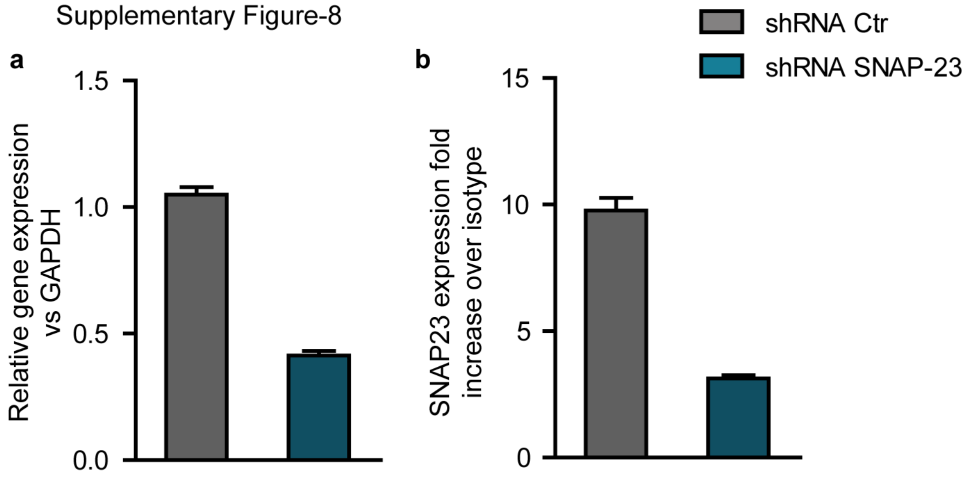

Supplementary Figure 8. **Reduction of SNAP-23 expression in melanoma cells transfected with SNAP-23 shRNA. a**) Reduced SNAP-23 gene expression as detected by RT-PCR. **b**) Reduced SNAP-23 protein expression as detectd by FACS analysis using an anti-SNAP-23 antibody. Results are from one representative experiment out of two performed in triplicates.

Supplementary Figure-9

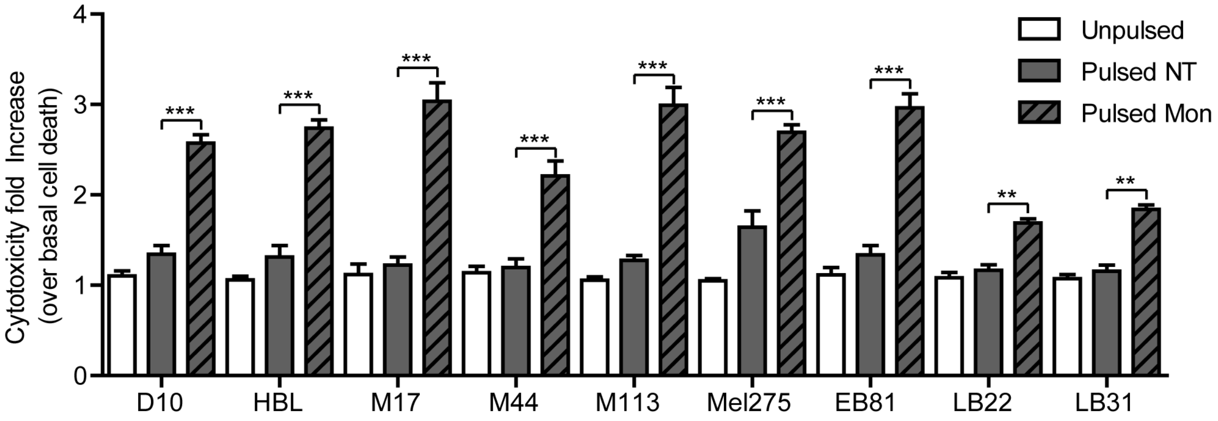

Supplementary Figure 9**. Treatment with monensin enhances CTL-mediated cytotoxicity in nine melanoma cell lines.** Melanoma either untreated (NT) or pretreated for 2 hours with monensin (Mon) were conjugated with specific CTL for 4 hours. Cells were either unpulsed of pulsed with 10µM antigenic peptide. Cytotoxicity is reported as fold increase over basal cell death. Results are expressed as mean ± SEM of 3 independent experiments. Two-Way Anova test using the GraphPad Prism software (version 6; GraphPad) was used to determine the statistical significance of differences between the groups. ***$P<0.001$; **$P<0.01$.

Supplementary Figure-10

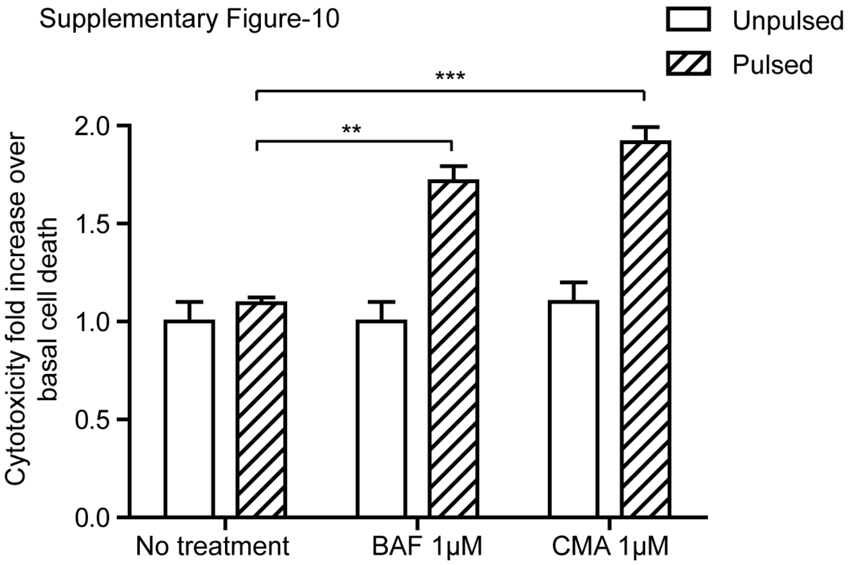

Supplementary Figure 10**. Treatment of melanoma cells with bafilomycin A1 or concanamycin A enhances CTL-mediated cytotoxicity.** Melanoma cells either untreated or pretreated for 2 hours with bafilomycin A1 (BFA) or concanamycin A (CMA) were conjugated with specific CTL for 4 hours. Cells were either unpulsed of pulsed with 10µM antigenic peptide. Cytotoxicity is reported as fold increase over basal cell death. Results are expressed as mean ± SEM of 3 independent experiments. Unpaired Student's t-test using the GraphPad Prism software (version 6; GraphPad) was used to determine the statistical significance of differences between the groups. ***P<0.001; **P<0.01.

Supplementary Figure-11

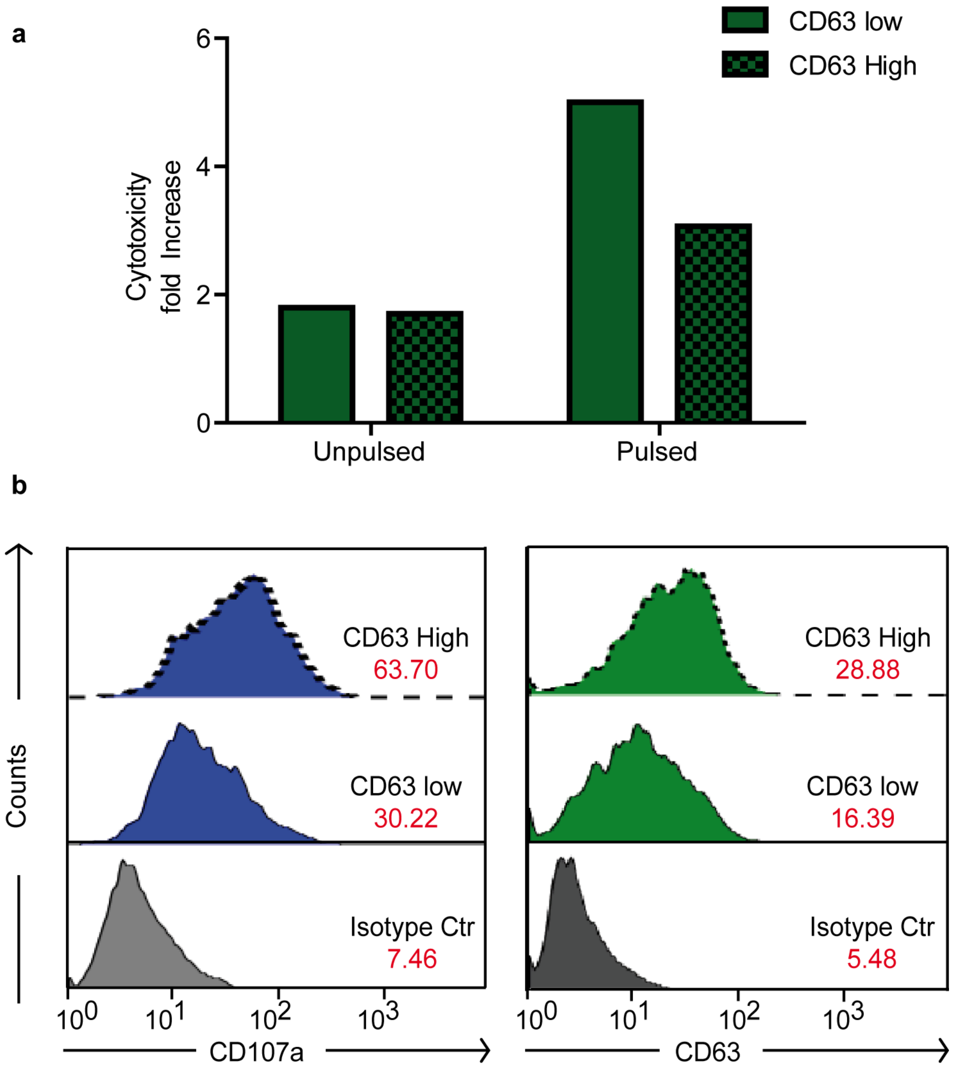

Supplementary Figure 11. **The resistance of conventional target cells to CTL-mediated cytotoxicity correlates with the level of CD63 surface expression. a)** JY cells previously sorted on the level of expression of CD63 were pulsed or not with antigenic peptide and co-incubated with CTL for 4 hours. Cytotoxicity was evaluated by FACS analysis using 7-AAD uptake and is reported as fold increase over basal cell death. **b)** To evaluate the level of LLE markers expression by cells used in a), the intracellular expression of CD107a and CD63 of sorted cells was measured by FACS analysis. Numbers indicate median fluorescent intensity (MFI). Data are from one representative experiment out of two.
